# Mutational analysis of the mitotic exit GTPase MoTem1 reveals its role in development, stress adaptation, pathogenicity and global gene regulation in *Magnaporthe oryzae*

**DOI:** 10.1007/s44154-026-00310-8

**Published:** 2026-05-09

**Authors:** Mengtian Pei, Xuze Xie, Yingying Cao, Jia Chen, Fan Yang, Zonghua Wang, Stefan Olsson, Guo-dong Lu, Ya Li

**Affiliations:** 1 State Key Laboratory of Agricultural and Forestry Biosecurity, College of Plant Protection, Fujian Agriculture and Forestry University, Fuzhou, 350002 China; 2 Fujian Key Laboratory of Aptamers Technology, Fuzong Clinical Medical College of Fujian Medical University (900th Hospital), Fuzhou, 350002 China; 3 Fuzhou University Affiliated Provincial Hospital, School of Medicine, Fuzhou University, Fuzhou, 350002 China; 4 Synthetic Biology Center, College of Future Technologies, Fujian Agriculture and Forestry University, Fuzhou, 350002 China

**Keywords:** Mitotic exit network, Pathogenicity, Stress, Tem1, Chitin synthase, RNA-Seq, *Magnaporthe oryzae*

## Abstract

The mitotic exit network (MEN), regulated by the small GTPase Tem1, plays a crucial role in coordinating cytokinesis and cell cycle progression in eukaryotes. In this study, we identified MoTem1, a functional homolog of Saccharomyces cerevisiae Tem1, in the rice blast fungus Magnaporthe oryzae, and investigated its role in mitotic regulation and pathogenesis. Using targeted mutagenesis, we generated a series of mutant strains: Δ*Motem1* (knockout), *MoTem1-OE* (overexpression), as well as *MoTem1-CA* (constitutively active) and *MoTem1-DN* (dominant-negative) variants created via single-nucleotide substitutions. Phenotypic characterization revealed that MoTem1’s activity states are critical for fungal growth, development, stress tolerance, and pathogenicity. While Δ*Motem1* and *MoTem1-CA* strains showed reduced virulence, the *MoTem1-DN* mutant exhibited hypervirulence. Transcriptomic profiling and weighted gene co-expression network analysis (WGCNA) identified chitin synthase *MoCHS1* as a downstream gene whose expression is directly or indirectly influenced by MoTem1 activity states. Pharmacological inhibition of chitin synthesis using Polyoxin B in *MoTem1-CA* showed increased sensitivity, confirming a decreased expression of chitin synthase in the *MoTem1-CA* strain. Subcellular localization studies revealed GTP-dependent spindle pole body (SPB) targeting, with inactive MoTem1 failing to localize to SPBs, while constitutive MEN activation in *MoTem1-CA* disrupted spindle position checkpoint (SPOC) controls, resulting in multinucleate hyphae and a range of developmental defects. In conclusion, our work establishes MoTem1 not merely as a cell cycle regulator, but as a global upstream factor that influences nuclear division, cell wall integrity, and broadly reshapes the genomic regulatory network to govern development and pathogenesis in *M. oryzae*.

**Supplementary Information:**

The online version contains supplementary material available at 10.1007/s44154-026-00310-8.

## Introduction

In eukaryotic organisms, the cell cycle is divided into the interphase and the mitotic phase (M-phase), with the latter consisting of both nuclear division (mitosis) and cytoplasmic separation (cytokinesis) (Nigg 2001). The spatiotemporal regulation of mitotic exit and the initiation of cytokinesis is controlled by the evolutionarily conserved mitotic exit network (MEN), a signaling cascade critical for coupling nuclear division to cell division fidelity (Bardin and Amon 2001; Hotz and Barral 2014). In *Saccharomyces cerevisiae*, MEN functions as a GTPase-driven signaling hierarchy centered on the Ras-like GTPase Tem1 (Termination of M-phase Protein 1), which orchestrates downstream effectors including Bfa1, Bub2, Cdc5, Lte1, Cdc15, Dbf2, Mob1, and the protein phosphatase Cdc14 (Bettignies and Johnston 2003). A key component of this process is the Bfa1-Bub2 heterodimer, which acts as a GTPase-activating protein (GAP) complex that hydrolyzes GTP bound to Tem1. This mechanism maintains MEN signaling in a spatially confined inactive state during early mitosis (Scarfone and Piatti 2015). MEN activation hinges on antagonistic phosphorylation events since the polo-like kinase Cdc5 phosphorylates Bfa1-Bub2 during anaphase, inhibiting their GAP activity and stabilizing Tem1 in its active GTP-bound conformation (Scarfone and Piatti 2015). This enables Tem1 to activate the kinase cascade involving Cdc15 and Dbf2-Mob1, ultimately leading to the release of Cdc14 phosphatase from the nucleolus (Scarfone and Piatti 2015). Cdc14 facilitates mitotic exit by dephosphorylating substrates that promote B-type cyclin proteolysis and activate Sic1, a cyclin-dependent kinase (CDK) inhibitor (Scarfone and Piatti 2015). Spatial surveillance mechanisms ensure MEN fidelity. Improper spindle orientation or cytoskeletal defects trigger Kin4 kinase activation via a Lte1-mediated relief of Kin4 autoinhibition (Scarfone and Piatti 2015). Kin4 phosphorylates Bfa1-Bub2 to counteract Cdc5-mediated inhibitory modifications, thereby restoring GAP activity to inactivate Tem1 and block MEN signaling—a checkpoint preventing untimely mitotic exit under error conditions (Scarfone and Piatti 2015).

Thus, Tem1, a small GTPase localized to the spindle pole body (SPB), serves as the primary regulatory GTPase of the mitotic exit network (MEN), playing a crucial role in controlling mitotic progression and exit in eukaryotes (Valerio-Santiago and Monje-Casas 2011). Tem1 was originally identified through genetic screens for mitotic termination defects (Shirayama and Matsui 1994). Tem1 orthologs have been found to have conserved functional roles across phylogenetically diverse fungi. In *Schizosaccharomyces pombe*, the Tem1 homolog Spg1 (septum-promoting GTP-binding protein 1) orchestrates mitotic exit, cytokinesis, and septation (Schmidt et al. 1997). Similarly, *Candida albicans* CaTem1 is indispensable for mitotic exit and cytokinesis initiation. Its deletion leads to hyperpolarized growth, cytokinesis failure, and aberrant cell cycle reentry post-nuclear division (Milne et al. 2014).

Tem1 activity homeostasis seems critical for fungal pathogenicity: In *Colletotrichum orbiculare*, CoBfa1/CoBub2 knockout strains exhibit nonpathogenic phenotypes due to constitutive CoTem1 activation and premature mitotic exit (Fukada and Kubo 2015). Pathogenicity is, however, rescued upon reintroducing a GTPase-deficient mutant CoTem1 (G20V), confirming that dynamic cycling between GTP- and GDP-bound states is essential for virulence (Fukada and Kubo 2015). Notably, *Fusarium graminearum* Δ*Fgtem1* demonstrates normal vegetative growth, sexual reproduction, and deoxynivalenol (DON) biosynthesis but displays severely impaired infection structure morphogenesis and significantly reduced pathogenicity on wheat spikes, implicating FgTem1 as a regulator important for pathogenesis (Miao et al. 2023).

Rice blast, caused by the fungus *Magnaporthe oryzae*, poses a significant global threat to rice, leading to annual yield losses that could feed 60 million people (Pennisi 2010; Dean et al. 2012). Under favorable conditions, *M. oryzae* develops conidiophores producing asexual conidia (Ou 1980). These three-compartment conidia with one nucleus in each compartment germinate on rice leaf surfaces, forming a single germ tube that develops into an appressorium that firmly attaches to the rice cuticle. The appressorium generates high turgor pressure, enabling the fungus to penetrate the rice cells using an infection peg that is forced through the leaf cuticle from an appressorium pore facing the leaf surface (Ou 1980). Once the cuticle is breached, a slender penetration peg delivers one nucleus, forming infection hyphae into the epidermal cells (Ou 1980). As a hemi-biotrophic pathogen, *M. oryzae* initially behaves biotrophically, hiding from the plant immune system before switching to necrotrophic growth (Wilson 2021). During the biotrophic phase, the fungus disrupts the hormone balance within the host and secretes effector proteins to evade immune responses, facilitating its spread through plasmodesmata between adjacent leaf cells (Kankanala et al. 2007; Fernandez and Orth 2018). Functional analyses of MEN components in the rice blast fungus *M. oryzae* have revealed their specialized roles in development and pathogenesis. MoBub2 is indispensable for conidiogenesis and appressorium differentiation; Δ*Mobub2* mutants produce conidia with hyper-septation and multinucleate compartments, coupled with appressorial defects that abolish host cuticle penetration (Fukada et al. 2019). Disruption of downstream MEN effectors, including MoSep1 (Cdc15 ortholog), MoMob1, and MoDbf2, severely attenuates pathogenicity and perturbs hyphal septation, resulting in elongated multinuclear hyphal segments (Feng et al. 2022). Intriguingly, *M. oryzae* exhibits evolutionary divergence in MEN signaling: Unlike in *S. cerevisiae* Cdc15, which phosphorylates Dbf2, MoSep1 directly activates MoMob1 via phosphorylation, bypassing MoDbf2 (Feng et al. 2022). Furthermore, deleting the MEN phosphatase MoCdc14 orthologue to phosphatases responsible for the final step in the cell cycle (Meitinger et al. 2012) regulates septal biogenesis, nuclear partitioning, and affect virulence by generating aseptate or hypo-septate multinucleate conidia forming nonfunctional appressoria that fail to breach plant epidermal barriers, underscoring MoCdc14's pleiotropic roles in infection (Li et al. 2018).

While homologs of the yeast Tem1-associated GTPase-activating protein (GAP) complex and downstream MEN components have been extensively characterized in *M. oryzae*, where they regulate septation and virulence, the absence of a confirmed Tem1 ortholog in this pathogen has hindered a comprehensive understanding of MEN signaling. Given the pivotal role of Tem1 as the master regulator of MEN in *S. cerevisiae*, where it orchestrates mitotic exit and ensures cell cycle fidelity, identifying its counterpart in *M. oryzae* is essential for elucidating how this pathway influences both fungal development and host infection. In this study, we identify MoTem1 (MGG_04862) as the functional Tem1 homolog in *M. oryzae* and investigate its regulatory roles through mutational and phenotypic analyses. Functional characterization revealed that MoTem1, with its GTPase activity, acts as a molecular switch that orchestrates mitosis, conidial morphogenesis, and affects stress adaptation, having indirect or direct effects on pathogenicity. Furthermore, we demonstrate that MoTem1 activities affect chitin synthase gene expression, influencing cell wall integrity and host invasion. Our findings establish MoTem1 as a crucial component of MEN signaling in *M. oryzae*, bridging mitotic regulation with growth and development as well as with reactions to biotic and abiotic environmental stresses.

## Results

### Identification of motem1 and generation and validation of MoTem1 functional Alleles, MoTem1^Q182L^ and MoTem1^T137N^ in *M. oryzae*

We identified a putative Tem1 orthologue in *M. oryzae* by a BLASTP search against the *M. oryzae* 70–15 genome (NCBI taxid: 242507) using *S. cerevisiae* Tem1 (ScTem1; NP_013647.1) as well as the more closely related *F. graminearum* FgTem1 (Miao et al. 2023) as queries. This analysis identified MGG_04862, annotated as a septum-promoting GTP-binding protein 1 at NCBI, which shares 60.21% sequence identity and 77% coverage with ScTem1, confirming its structural homology, and we thus designate it MoTem1 (Fig. S1A). The similarity with the more closely related *F. graminearum* FgTem1 (Miao et al. 2023) was even better, 72.28% sequence identity and 96% coverage.

**Fig. 1:**
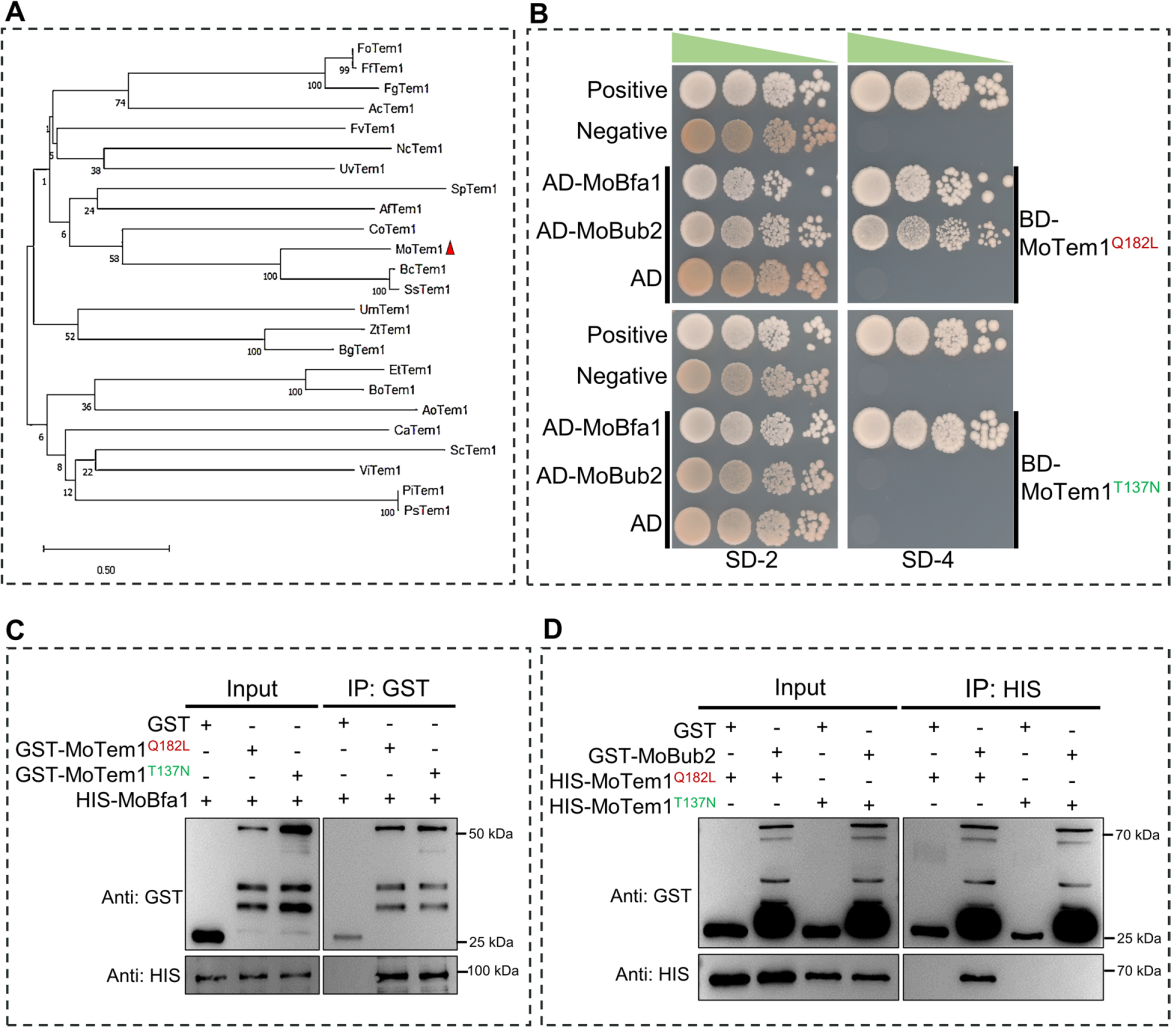
Identification of MoTem1 and verification of its interactions with orthologous proteins via yeast two-hybrid and pull-down assays. **A** Phylogenetic analysis of Tem1 homologs. A total of 24 Tem1 homologs were analyzed. Species information and accession numbers are provided in Table S1. **B** Yeast two-hybrid assay validating the interaction between MoTem1 and MoBfa1/MoBub2. MoTem1^Q182L^ represents a constitutively active form, whereas MoTem1^T137N^ denotes a dominant-negative form. Different states of BD-MoTem1 plasmids were co-transformed with AD-MoBfa1 or AD-MoBub2 plasmids into yeast AH109. Yeast growth in SD-2/-Tep/-Leu2 medium indicates successful plasmid transformation, while growth in SD-4/-Ade/-His/-Leu/-Trp medium suggests an interaction between proteins on AD and BD plasmids. The combination of AD-T and BD-P53 was used as a positive control, while AD-T and BD-Lam were used as a negative control. **C** Pull-down assay confirming the interaction between MoTem1 and MoBfa1. GST, GST-MoTem1^Q182L^, and GST-MoTem1^T137N^ were subjected to GST pull-down assays with HIS-MoBfa1, respectively, where the combination of GST and HIS-MoBfa1 was used as a negative control. **D** Pull-down assay validating the interaction between MoTem1 and MoBub2. Since HIS-MoBub2 could not be expressed, GST and GST-MoBub2 were used to pull down HIS-MoTem1^Q182L^ or HIS-MoTem1.^T137L^, testing the binding between different MoTem1 variants and MoBub2

Phylogenetic analysis of Tem1 orthologs from 24 fungal species resolved MoTem1 within a clade containing *Botrytis cinerea* BcTem1 and *Sclerotinia sclerotiorum* SsTem1, underscoring its evolutionary conservation among phytopathogenic fungi with heavily melanized propagules (Fig. 1A). To dissect the functional states of MoTem1, we introduced mutations to put the protein in active (Q182L) or inactive (T137N) states based on Tem1 homologs of *S. cerevisiae*, *C. orbicular,* and *F. graminearum* (Fig. S1B). For simplicity, we designated the active form of MoTem1 as MoTem1^Q182L^ and the inactive form as MoTem1^T137N^.

Yeast two-hybrid assays revealed that both MoTem1^Q182L^ and MoTem1^T137N.^ interact with MoBfa1 (MGG_03162), a core component of the GTPase-activating protein (GAP) complex. However, MoBub2 (MGG_04676), another core component of the GAP complex, binds exclusively to MoTem1^Q182L^ (Fig. 1B). Pull-down experiments corroborated these state-dependent interactions, demonstrating that MoTem1 ^Q182L^ recruits the full GAP complex (MoBfa1-MoBub2), while MoTem1^T137N^ engages only MoBfa1 (Fig. 1C, D).

**Fig. 2:**
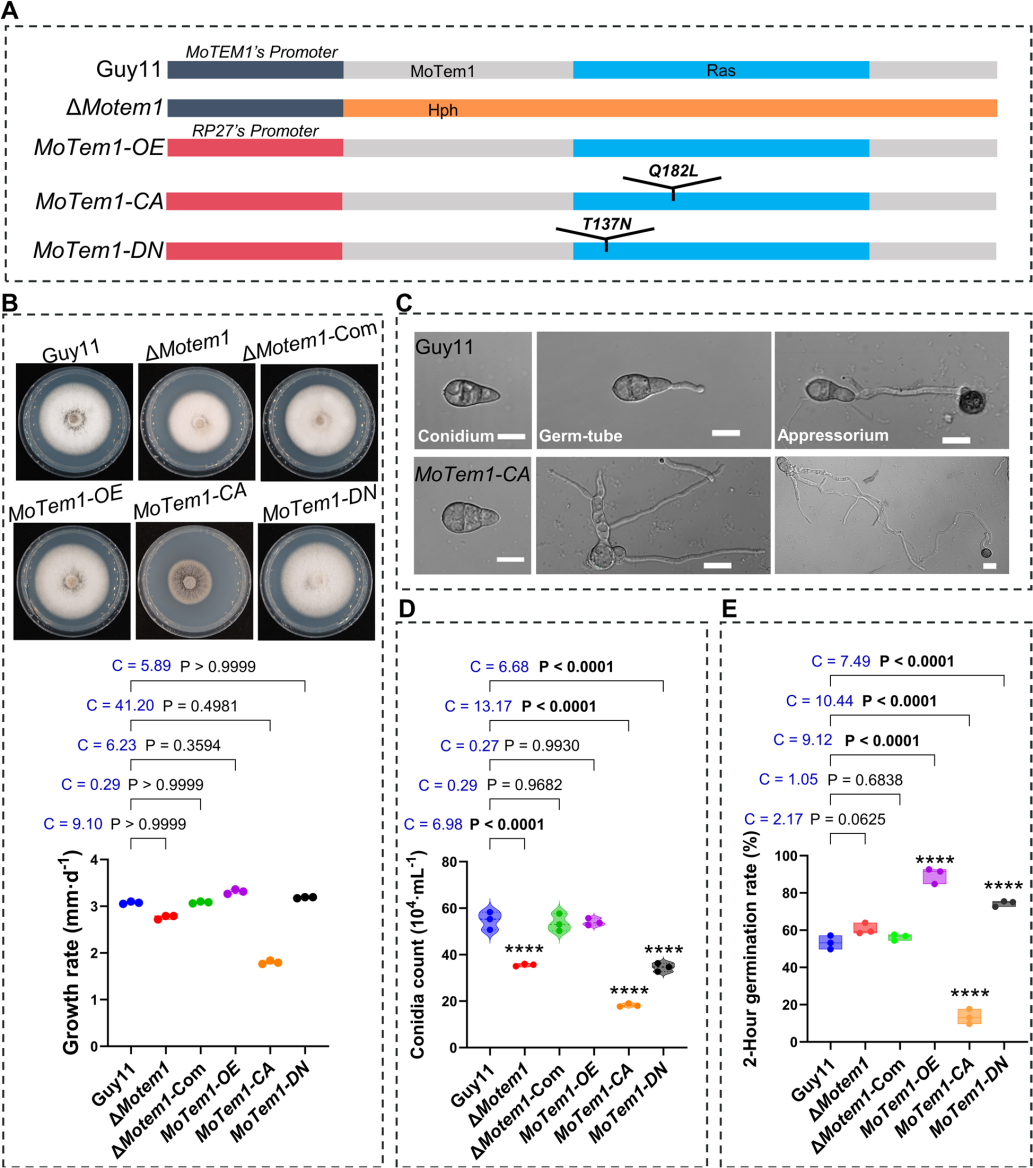
Creation of different mutant strains recording their phenotypes compared to WT. **A** Schematic diagram of the forms of MoTem1 expressed in different strains. In Guy11, *MoTEM1*’s promoter activates the *MoTEM1* gene transcription. In Δ*Motem1*, the *MoTEM1* gene has been replaced by the HYH gene; therefore, *MoTEM1*’s promoter activates HPH transcription. In *MoTem1-OE*, *MoTem1-CA*, and *MoTem1-DN* strains, the *MoTEM1*, *MoTEM1-Q182L*, and *MoTEM1-T137N* genes were behind the strong promoter RP27 and were transferred into Guy 11 to obtain *MoTem1-OE/CA/DN* strains. Driven by the RP27 promoter, overexpression of MoTem1, MoTem1^Q182L^, or MoTem1^T137N^ will override the function of the native MoTem1 expressed by Guy11. The gray and blue bar represents the amino acid length of MoTem1, the orange bar represents the amino acid length of HPH, the black bar represents the nucleotide length of the *MoTEM1* promoter, and the red bar represents the nucleotide length of the RP27 promoter. **B** Growth of different strains on solid CMII medium, including colony growth rate analysis. Statistical significance was determined by Kruskal–Wallis test with Dunn’s post hoc test. data represent means ± SD, n = 3 biological replicates. **C** Comparative observation of conidial germination between Guy11 and MoTem1-CA strains. Scale bars represent 10 μm. **D** Conidia production by the different strains. Statistical significance was determined by one-way ANOVA with Dunnett’s post hoc test. data represent means ± SD, *n* = 3 biological replicates. **E** Conidia germination rate after 2 h for the different strains. Statistical significance was determined by one-way ANOVA with Dunnett’s post hoc test. data represent means ± SD, n = 3 biological replicates. For B, D, E, Statistical significance compared to Guy11 is indicated as follows: *p < 0.05, **p < 0.01, ***p < 0.001, and ****p < 0.0001. Cohen’s d (C) is reported as a measure of effect size, with thresholds defined as: 0.2 = small, 0.5 = medium, and 0.8 = large

We consequently generated four isogenic mutant strains in the Guy11 background to investigate the functional consequences of MoTem1 activity states in *M. oryzae*: Δ*Motem1* (gene deletion), *MoTem1-OE* (overexpression), *MoTem1-CA* (Q182L; constitutively active), and *MoTem1-DN* (T137N; dominant-negative) (Fig. 2A). We replaced the *MoTEM1* coding sequence by homologous recombination with a hygromycin phosphotransferase (*HPH*) cassette to construct the Δ*Motem1* strain. Southern blot analysis using a *Hin*d III-digested genomic DNA probe confirmed the *MoTEM1* precise locus replacement, with a single integration event observed in the transformants (Fig. S1C). The *MoTem1-OE*, *MoTem1-CA*, and *MoTem1-DN* strains were all generated by transforming Guy11 with pKNT-RP27 vectors expressing wild-type *MoTEM1*, *MoTEM1-*Q182L, and *MoTEM1-*T137N alleles under the constitutive RP27 overexpression promoter. RT-qPCR quantification revealed 13.57 ± 1.77, 11.11 ± 1.91, and 19.78 ± 1.82-fold upregulation of *MoTEM1* transcripts in *MoTem1-OE*, *MoTem1-CA*, and *MoTem1-DN*, respectively, compared to wild-type (Fig. S1D).

Our results positively identified a *MoTem1* and established a panel of *M. oryzae* strains with defined MoTem1 activity states, enabling further systematic dissection of the roles of these states in the biology of *M. oryzae* growing as a pathogen, as well as growing on lab media.

### MoTem1 governs vegetative growth and conidiation through GTPase activity cycling

To assess the role of MoTem1 in fungal development, we measured the mycelial radial growth of each strain. Compared with the wild type (WT, Guy11; 3.08 ± 0.02 mm/d), the growth of Δ*Motem1* (2.77 ± 0.04 mm/d) and *MoTem1-CA* (1.80 ± 0.04 mm/d) decreased by approximately 10.06% and 41.56%, respectively, while that of *MoTem1-OE* (3.31 ± 0.05 mm/d) and MoTem1-DN (3.19 ± 0.01 mm/d) increased by about 7.41% and 3.57%, respectively. Although the Kruskal–Wallis test with Dunn’ s post-hoc test indicated that none of these differences reached statistical significance (Fig. 2A, B), effect size (Cohen’ s d) analysis revealed clear trends. According to the conventional interpretation (d ≥ 0.8 representing a large effect), the effect size for *MoTem1-CA* relative to WT was extremely large (d = 41.22). Furthermore, the effect sizes for Δ*Motem1* (d = 9.1), *MoTem1-OE* (d = 6.27), and *MoTem1-DN* (d = 5.85) also far exceeded the threshold for a “large effect,” suggesting that the growth alterations observed in these strains reflect considerable phenotypic magnitudes.

Conidiation assays on rice bran medium revealed severe defects in asexual reproduction. Compared to the wild type, the Δ*Motem1*, *MoTem1-CA*, and *MoTem1-DN* strains produced 35.09%, 66.74%, and 36.92% fewer conidia, respectively (p < 0.0001 for all comparisons, one-way ANOVA with Dunnett’ s post hoc test; Fig. 2D). The magnitude of these reductions was supported by very large effect sizes (Cohen’ s d: 6.98, 13.17, and 6.69, respectively). In contrast, conidiation in the *MoTem1-OE* strain was comparable to WT levels, indicating that MoTem1 overexpression alone does not affect this process. These results demonstrate that the GDP/GTP cycling of MoTem1-rather than its expression level per se—is essential for conidiation, as both the locked-state (CA and DN) and knockout (Δ) mutants profoundly impair conidiation efficiency.

Collectively, MoTem1 operates as a regulator of fungal development: its GTPase activity promotes vegetative growth, while dynamic GDP/GTP cycling is critical for conidia production.

### MoTem1 is involved in conidium germination and appressorium formation

To investigate the potential role of MoTem1 in pre-infection development, we analyzed conidial germination and appressorium formation in the mutant strains. Compared with the wild type (WT; 53.35 ± 3.67%), *MoTem1-OE* and *MoTem1-DN* exhibited significantly accelerated conidial germination, reaching 89.64 ± 4.27% and 74.38 ± 1.52%, respectively, at 2 h post-inoculation (hpi) (p < 0.0001 for *MoTem1-OE* and *MoTem1-DN* vs. WT, one-way ANOVA with Dunnett’ s post hoc test; Fig. 2E), with very large effect sizes (Cohen’ s d = 9.12 and 7.49). This accelerated phenotype correlated with their enhanced colony radial growth rates (Fig. 2B). In sharp contrast, MoTem1-CA showed severe and significantly delayed germination (only 13.47 ± 3.96% at 2 hpi; *p* < 0.0001 for MoTem1-CA vs. WT, one-way ANOVA with Dunnett’s post hoc test; Fig. 2E and Fig. S2B), accompanied by an extremely large effect size (Cohen’ s d = 10.44), and also displayed defects in appressorium formation (Fig. 2E and Fig. S2B). These results indicate that sustained GTPase activity impedes germination initiation.

Morphological analysis revealed that *MoTem1-CA* conidia exhibited aberrant phenotypes, including multiple germ tube formation and cell wall hypertrophy (Fig. 2C and Fig. S2A). Notably, *MoTem1-CA* appressoria formed abnormally elongated germ tubes, whereas other strains displayed WT-like germ tube-appressorium transitions (Fig. 2C and Fig. S2A). These defects suggest that persistent MoTem1 activation disrupts germ tube polarity coordination, while its inactivation permits a timely developmental progression.

These findings establish that MoTem1’s GTPase cycling, not merely its presence, orchestrates the spatiotemporal progression of conidial germination and appressorium differentiation processes that are critical for normal plant pre-penetration morphogenesis, and is necessary for the formation of appressoria and pathogenicity.

### MoTem1 activity states modulate *M. oryzae* pathogenicity

**Fig. 3:**
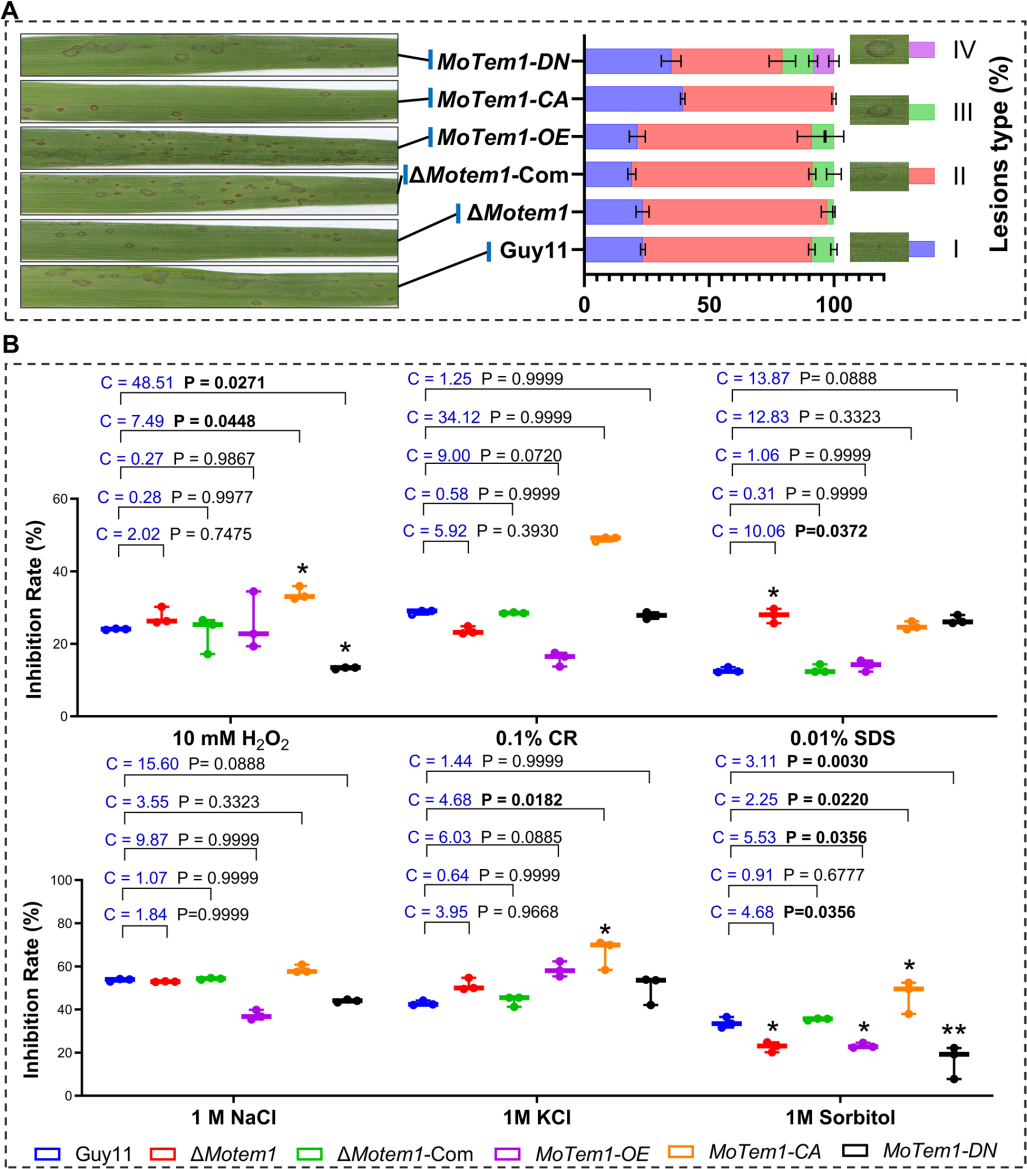
Motem1 involvement in stress response and pathogenic process. **A** Lesion types in rice leaves caused by different strains. Lesions less than 0.05 cm in length are classified as type 1, those 0.05–0.2 cm as type 2, 0.2–0.3 cm as type 3, and 0.3–0.4 cm as type 4 (*n* = 3 biological replicates, data are means ± SD). Color enhancement of lesion colors to show lesions better. The original images can be seen in Fig. S3. **B** Inhibition rate of different strains under oxidative stress (H_2_O_2_, cell wall stress (CR), and membrane stress (SDS) ionic + osmostic stress of non-nutrient NaCl and nutrient KCl, and osmotic stress of non-nutrient (Sorbitol). For H_2_O_2_ and Sorbitol, Statistical significance was determined by one-way ANOVA with Dunnett’s post hoc test. data represent means ± SD, *n* = 3 biological replicates. For CR, SDS, NaCl and KCl, Statistical significance was determined by one-way ANOVA with Dunnett’s post hoc test. data represent means ± SD, *n* = 3 biological replicates. Statistical significance compared to Guy11 is indicated as follows: *p < 0.05, **p < 0.01, ***p < 0.001, and ****p < 0.0001. Cohen’s d (C) is reported as a measure of effect size, with thresholds defined as: 0.2 = small, 0.5 = medium, and 0.8 = large

We performed spray inoculations of rice seedlings (cv. CO39) with conidia from mutant strains and quantified lesion progression using a five-tier grading system to directly assess MoTem1’s role in rice plant infection. WT infections produced Type I (23.46 ± 0.78%), II (67.71 ± 1.07%), and III (8.83 ± 1.04%) lesions, consistent with its virulence profile (Fig. 3A and Fig. S2). *MoTem1-OE* recapitulated this pattern (Type I: 21.29 ± 2.62%, Type II: 69.74 ± 4.68%, Type III: 8.97 ± 3.18%), suggesting little effect of the overexpression or that functional compensation or feedback regulation preserves pathogenicity (Fig. 3A).

Δ*Motem1* infections exhibited attenuated pathogenic lesion size growth, marked by a 3.2-fold reduction in Type III (2.76 ± 0.37%) lesions and a proportional shift to Type II (73.89 ± 1.87%) lesions. Strikingly, *MoTem1-CA* infections were restricted to Type I (39.49 ± 0.73%) and II (60.51 ± 0.73%) lesions, indicating that persistent GTPase activity severely compromises virulence (Fig. 3A and Fig. S3). Conversely, *MoTem1-DN* displayed hypervirulence, generating Type IV lesions (8.28 ± 1.64%) absent in WT at the same HPIs, alongside elevated Type III (12.38 ± 1.45%) and reduced Type II (44.53 ± 4.30%) frequencies (Fig. 3A).

These results establish *MoTem1* as a tunable regulator of growth and nuclear division outcomes: its dynamic inactivation promotes more aggressive pathogenesis, while constitutive activation or deletion reduces virulence, likely through dysregulated effector deployment or out-of-time morphogenic coordination.

### MoTem1 affects stress adaptation through single nucleotide-dependent signaling

We challenged strains with oxidative, cell wall, and osmotic stressors mimicking host defense conditions that the fungus must be able to handle in its natural habitat as a plant pathogen to assess MoTem1’s role in environmental stress resilience.

To evaluate the impact of MoTem1 on oxidative stress tolerance, we assessed the growth of mutant strains under 10 mM H_2_O_2_. Compared to the wild type (WT; growth inhibition rate of 11.13 ± 1.43%), *MoTem1-OE* and *MoTem1-DN* exhibited enhanced tolerance (inhibition rates of 7.15 ± 2.00% and 5.33 ± 2.28%, respectively). The increased tolerance in *MoTem1-DN* was statistically significant (*p* = 0.0271 vs. WT, one-way ANOVA with Dunnett’ s post hoc test) and accompanied by an extremely large effect size (Cohen’ s d = 48.51). In contrast, Δ*Motem1* and *MoTem1-CA* showed hypersensitivity to oxidative stress (inhibition rates of 14.95 ± 2.06% and 26.39 ± 4.84%, respectively), with the increased sensitivity in *MoTem1-CA* also being significant (*p* = 0.0448 vs. WT) and supported by a large effect size (Cohen’s d = 7.49). These phenotypic trends were further corroborated by half-maximal inhibitory concentration (IC_50_) analysis: the IC_50_ for *MoTem1-DN* (15.48 mM) was higher than that of the WT (11.47 mM), whereas the IC_50_ for *MoTem1-CA* (10.79 mM) was lower. These results indicate that disrupting the GTP/GDP cycle of MoTem1 (DN state) enhances oxidative stress tolerance, while its constitutive activation (CA state) increases fungal susceptibility (Fig. 3B and Fig. S4).

For cell wall integrity and membrane permeability stresses, the mutants showed distinct phenotypic profiles in response to 0.1% Congo Red (CR) and 0.01% SDS. Under CR stress, *MoTem1-OE* exhibited markedly lower growth inhibition (15.92 ± 1.93%) compared to the wild type (WT; 28.76 ± 0.59%), supported by a large effect size (Cohen’ s d = 9.0), though the difference was not statistically significant (p > 0.05, Kruskal–Wallis test with Dunn’ s post hoc test, Fig. 3C and Fig. S4). In contrast, *MoTem1-CA* displayed severe hypersensitivity to CR (48.93 ± 0.59% inhibition; Cohen’ s d = 34.12). Corresponding IC_50_ values were consistent with these trends: *MoTem1-OE* (0.196%) showed higher tolerance than WT (0.184%), whereas *MoTem1-CA* (0.115%) was more sensitive (Fig. 3C and Fig. S4).

Under SDS stress, ΔMotem1 (27.78 ± 1.99% inhibition) was significantly more sensitive than WT (12.72 ± 0.73%; *p* = 0.0372, Kruskal–Wallis test with Dunn’ s post hoc test), with a very large effect size (Cohen’ s d = 10.06). MoTem1-CA (24.94 ± 1.13%) and *MoTem1-DN* (26.59 ± 1.21%) also exhibited higher inhibition rates than WT, though these differences were not statistically significant (*p* > 0.05). *MoTem1-OE* (14.00 ± 1.55%) showed an inhibition rate similar to WT. IC_50_ analysis further supported these observations: ΔMotem1 (0.01291%), *MoTem1-CA* (0.01243%), and *MoTem1-DN* (0.01275%) all had lower IC_50_ values than WT (0.01536%), whereas *MoTem1-OE* (0.01574%) was comparable to WT (Fig. 3C and Fig. S4). Together, these data indicate that MoTem1 distinctly modulates cell wall and membrane integrity in an activation-state-dependent manner: overexpression confers CR-specific tolerance, constitutive activation causes severe hypersensitivity to CR, and loss of MoTem1 or locked-inactive states increase susceptibility to SDS-mediated membrane stress.

Under ionic and osmotic stress conditions (1 M NaCl, 1 M KCl, 1 M sorbitol), the mutants exhibited allele-specific responses (Fig. 3B and Fig. S3). *MoTem1-CA* showed consistently high sensitivity across all stresses, with significantly increased inhibition under KCl stress (66.41 ± 7.02% vs. WT 42.88 ± 1.13%; Cohen’ s d = 4.68) and a lower IC_50_ for NaCl (0.71 M vs. WT 0.98 M). In contrast, ΔMotem1, *MoTem1-OE*, and *MoTem1-DN* displayed enhanced tolerance to sorbitol (inhibition rates of 22.68 ± 2.33%, 23.19 ± 1.20%, and 16.39 ± 7.58%, respectively, vs. WT 33.87 ± 2.45%; Cohen’ s d = 4.68, 5.53, and 3.11), whereas they showed varied responses to ionic stresses: *MoTem1-OE* and *MoTem1-DN* had slightly higher NaCl IC_50_ values (1.03 M and 1.04 M) than WT, but *MoTem1-OE* exhibited increased inhibition under KCl (58.54 ± 3.49%; Cohen’ s d = 6.03). These results indicate that constitutive MoTem1 activation broadly increases stress sensitivity, while its overexpression or loss-of-function promotes osmotic tolerance but differentially affects ion stress responses, suggesting that MoTem1 regulates distinct pathways for adapting to membrane and osmotic.

Collectively, MoTem1 affects downstream stress signaling: its GTP-bound state promotes oxidative resilience and osmotic adaptation, while dynamic GTP/GDP cycling maintains fungal cell wall and membrane functions.

### Nucleotide mutation dependent SPB-localization of MoTem1 coordinates mitotic fidelity

**Fig. 4:**
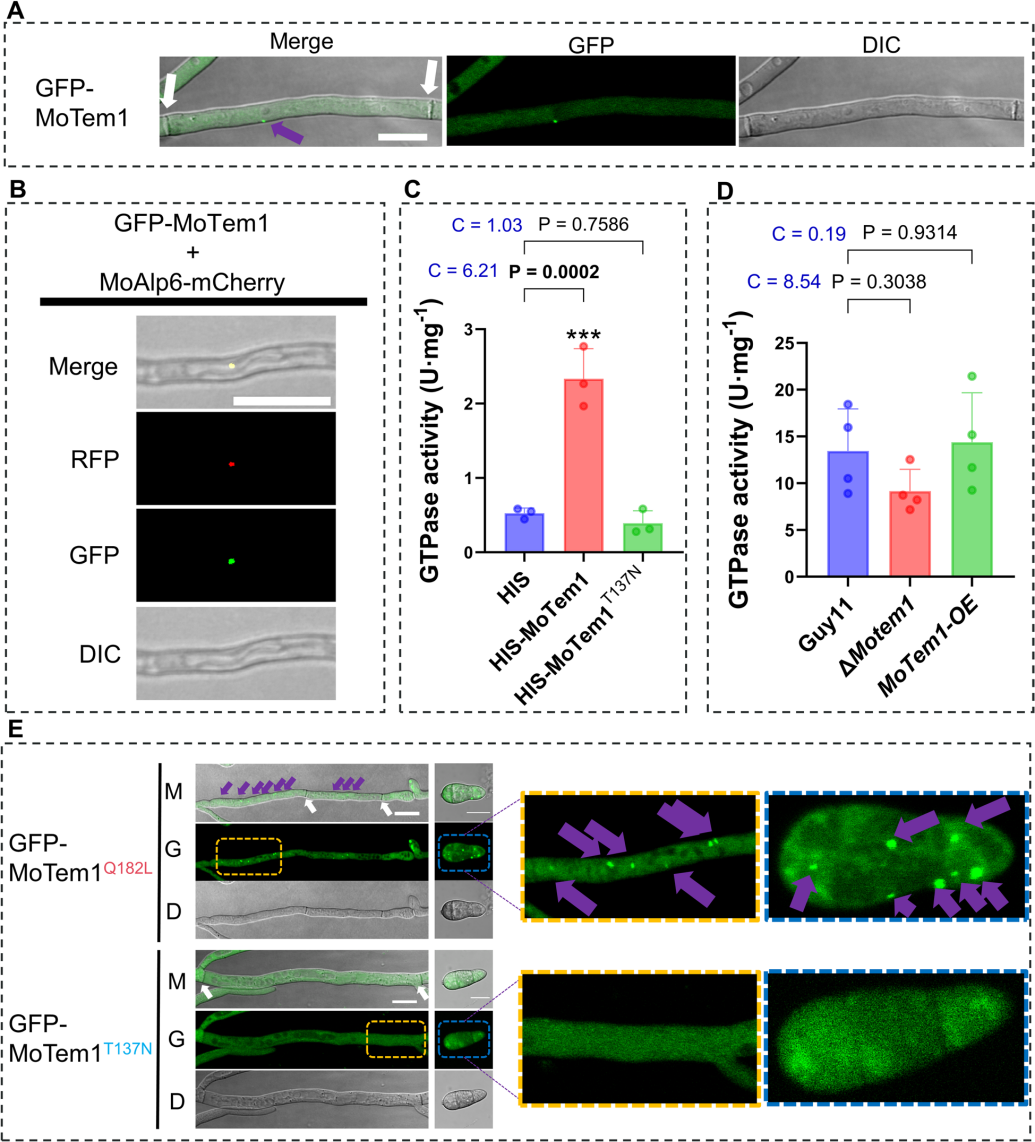
Motem1 gtpase activity modulates its localization to the spindle pole body. **A** Subcellular localization of MoTem1 to the spindle pole body (SPB) like structure in Guy 11 hyphae (WT). White size bars in the Merge images represent 10 μm, and white arrows mark the position of septa in hyphal compartments, and blue arrows mark the spindle pole body (SPB) like structure positions in hyphae. **B** Subcellular localization of MoTem1. MoAlp6 (MGG_01815) is located in the spindle and serves as a spindle pole body marker. Scale bar, 10 μm. **C** GTPase activity of MoTem1 is much higher than that of MoTem1^T137N^ and shows the deactivation of MoTem1 GTPase by the T137N substitution. Statistical significance was determined by one-way ANOVA with Dunnett’s post hoc test. data represent means ± SD, *n* = 3 biological replicates. **D** GTPase activity of *ΔMotem1* is slightly but not significantly reduced compared to Guy11 and the MoTem1-OE strains that show similar activities. Statistical significance was determined by one-way ANOVA with Dunnett’s post hoc test. data represent means ± SD, *n* = 4 biological replicates. For C and D, Statistical significance compared to HIS or Guy11 is indicated as follows: *p < 0.05, **p < 0.01, ***p < 0.001, and ****p < 0.0001. Cohen’s d (C) is reported as a measure of effect size, with thresholds defined as: 0.2 = small, 0.5 = medium, and 0.8 = large. **E** The subcellular localization of GFP- MoTem1^Q182L^, and GFP-MoTem1^T137N^ was observed in mycelia (left) and the 3-compartments of conidia (Right). Orange or blue dashed areas are shown magnified to the far right. G represents GFP fluorescence, D represents DIC microscopy, and M represents merged G and D. White size bars in the Merge images represent 10 μm, and white arrows mark the position of septa in hyphal compartments, and blue arrows mark the SPB-like positions in hyphae and conidia

Fluorescence localization using EGFP-MoTem1 in M. oryzae hyphae revealed a punctate localization pattern (Fig. 4A). Co-expression with the SPB marker MoAlp6-mCherry (expressed from the pKNT-RP27-mCherry vector) confirmed that GFP-MoTem1 specifically localizes to spindle pole bodies (SPBs) (Fig. 4B). To investigate the biochemical basis of this localization, we assessed the GTPase activity of MoTem1. In vitro, purified HIS-MoTem1 exhibited significant GTP-hydrolyzing activity (2.33 ± 0.40 U/mg), which was significantly higher than that of the HIS-tag control (0.53 ± 0.07 U/mg; p = 0.0002) with a very large effect size (Cohen’ s d = 6.21), whereas the inactive mutant HIS-MoTem1^T137N^ showed no significant increase in activity (0.39 ± 0.17 U/mg) (Fig. 4C). In vivo, while Δ*Motem1* showed a decreasing trend (9.16 ± 2.33 relative activity units) and *MoTem1-OE* an increasing trend (14.39 ± 5.29) compared to the wild type (13.45 ± 4.49), these differences were not statistically significant (Fig. 4D). Notably, the large effect size for Δ*Motem1* (Cohen’ s d = 8.54) being suggestive of a meaningful biological reduction (Fig. 4D). Consistent with these enzymatic properties, localization analysis of the functional mutants showed that the constitutively active MoTem1^Q182L^ displayed markedly intensified and more numerous SPB-associated puncta in both hyphae and conidia. In contrast, the dominant-negative mutant MoTem1^T137N^ completely lacked defined punctate localization, with fluorescence diffusely distributed throughout the cytoplasm (Fig. 4E). Taken together, the GTP-bound form of MoTem1 is enriched at SPBs, while the GDP-bound form is diffusely localized in the cytoplasm.

**Fig. 5:**
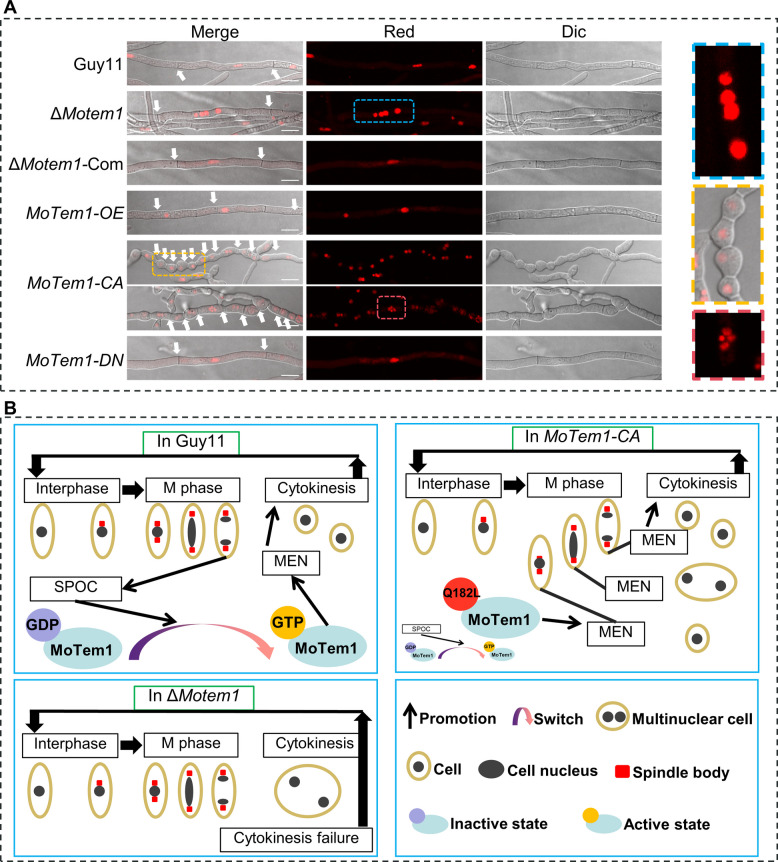
Effect of MoTem1 on nuclear distribution in relation to septation into separate hyphal compartments. **A** Guy11, *ΔMotem1-Com*, *MoTem1-OE,* and *MoTem1-DN* contain 1 nucleus in each normal-length hyphal compartment. *MoTem1-CA* also contains both single-nucleus and multi nucleus compartment, but these compartments are very short. *ΔMotem1* have normal-length compartments, but they are multinucleate, indicating failed cytokinesis. **B** Schematic representation of the observed nuclear distribution in relation to MoTem1 activities in Guy11, MoTem1‑CA, and ΔMotem1. In ΔMotem1, cytokinesis failure leads to multinucleated cells. In MoTem1‑CA, aberrant MEN signaling at different stages of mitosis causes premature cytokinesis, resulting in either multinucleated cells or smaller cells. Since residual WT MoTem1 activity is present in the strain overexpressing *MoTem1-DN* (current standard for these experiments) and not completely absent as in the deletion, this residual activity may be enough for normal cytokinesis

To visualize nuclei, we used the nuclear marker MoHis1-mCherry (MGG_12797) (Zhang et al. 2019) to resolve the different mutants MoTem1’s effect on subcellular nuclear dynamics. Consistent with a MEN pathway dysregulation, Δ*Motem1,* and *MoTem1-CA* hyphae harbor multinucleate hyphal compartments indicating septal formation and nuclear segregation not synchronized with nuclear division (Fig. 5A). In contrast, *MoTem1-DN* maintained WT-type hyphal septa formation and nuclear segregation, suggesting transient MEN inactivation preserves mitotic exit coordination with septal formation and nuclear migration. A schematic model summarizing the proposed mechanism by which the disruption (Δ*Motem1*) or constitutive activation (*MoTem1-CA*) of MoTem1 leads to multinucleated cells is presented in Fig. 5B, illustrating how aberrant GTPase cycling impacts nuclear division and cytokinesis.

Our data establish the MoTem1 regulator of the *M. oryzae* MEN pathway, where its SPB-localized GTP-bound state affects mitotic exit. It further shows that deletion (Δ*Motem1)* or constitutive activation of MoTem1 (*MoTem1-CA)* disrupts the connection between nuclear division and nuclear separation into separate hyphal compartments.

### Nucleotide-mutation states of MoTem1 change transcriptional regulatory networks in *M. oryzae*

To dissect the genome‑wide regulatory effects of MoTem1, we conducted RNA-Seq on Guy11, Δ*Motem1*, *MoTem1-OE*, *MoTem1-CA*, and *MoTem1-DN* strains. The absence of significant batch effects, as evidenced by consistent global expression distributions across all groups and the correlation heatmap among biological replicates demonstrated high reproducibility (Fig. S6A and S6B). Principal component analysis (PCA) clearly separated Δ*Motem1* and *MoTem1-CA* from Guy11 along the first principal component (PC1), while *MoTem1-OE* and *MoTem1-DN* clustered near Guy11, indicating pronounced transcriptional alterations in the deletion and constitutively active strains (Fig. S6C). Differential expression analysis was performed using DESeq2 with a stringent threshold (FDR < 0.01 and |log2FC|> 2). Venn analysis of the resulting differentially expressed genes (DEGs) identified 72 conserved DEGs across all mutant comparisons (Fig. S7A). These conserved genes include core cell-cycle regulators and stress-responsive transcription factors, which may underlie the phenotypic divergences observed in this study.

In Δ*Motem1*, downregulated genes were enriched for "glycogen metabolic process", "melanin metabolic process", consistent with observed growth retardation, attenuated virulence, and multinucleate phenotypes potentially resulting from disrupted fundamental metabolism functions (Fig. S7B, Table. S4).

*MoTem1-OE* specifically activates genes associated with "oxidoreductase activity" (Fig. S7C) whereas *MoTem1-DN* primarily upregulates genes involved in "primary metabolic processes". This demonstrates that both achieve the same stress-tolerant phenotype through distinct molecular pathways: one enhancing direct detoxification and the other promoting metabolic reorganization (Fig S7E, Table. S4). In *MoTem1-CA*, upregulated genes were prominently enriched in pathways including "single-organism metabolic process" and "response to stress", alongside substantial expression of genes related to "intrinsic component of membrane". This global transcriptional reprogramming suggests that cells activate broad metabolic, transport, and adaptive responses to cope with internal stresses, which may underlie the observed severe growth defects, multinucleation, and heightened stress sensitivity (Fig. S7D, Table S4). These transcriptional landscapes position MoTem1 as a signaling hub that influences responses through dynamic GTP/GDP nucleotide cycling with many effects on redox balance, pathogenicity, and morphogenesis, with regulatory effects reminiscent of MoIsw2 (Pei et al. 2024).

### Weighted co-expression network analysis identifies MoTem1-regulated functional modules

To resolve transcriptional networks underlying MoTem1-mediated phenotypes, we performed weighted gene co-expression network analysis (WGCNA), identifying eight distinct modules (Fig. S8 and S9).

Module-trait correlation analysis revealed three functional clusters of special interest for plant pathogenicity. The MEbrown genes were positively correlated with oxidative stress resistance (*r* = 0.91) and negatively with growth rate (*r* = −0.95), suggesting a growth-stress adaptation trade-off (Fig. S9). The MEyellow module showed a strong association with hyphal growth (*r* = 0.80) and was enriched in ribosome biogenesis and glycolytic genes (Fig. S9). The MEtan module exhibited the highest correlation with pathogenicity (*r* = 0.71) and contained 38 virulence-associated genes, including those encoding secreted effectors and cutinases (Fig. S9).

This network topology positions MEbrown and MEtan-regulated genes as important in stress adaptation and virulence, respectively. Three of the 72 DEGs responding genes in MEbrown, MGG_02246, MGG_11991, and MGG_06888 were selected for RNAseq versus RT-qPCR cross-validation. Similarly, for MEtan, MGG_01802, MGG_05421, and MGG_06238 were selected. The two methods show a similar expression pattern of responses of the five different strains for all genes, validating the RNAseq results (Fig. S11).

Expression dynamics validated these associations. MEbrown genes were strongly upregulated in *MoTem1*-*CA* and downregulated in the other strains (Fig. S12). MEyellow genes were suppressed in fast-growing Δ*Motem1* as in slow-growing *MoTem1-CA* but upregulated in slow-growing *MoTem1-DN* (Fig. S13). METan genes showed reduced expression in hypovirulent Δ*Motem1* and *MoTem1-CA* (Fig. S14), aligning with lesion severity metrics. Note: MEtan includes *MoCHS1* (MGG_01802), a chitin synthase possibly linking this module to cell wall-mediated plant immune responses.Taken together, the analysis further supports these effects along similar lines as for MoIsw2 epigenetic stress regulation (Pei et al. 2024) and, in addition, suggests specific effects on chitin synthesis that might explain some of the phenotypes.

### MoTem1 activity affects pathogenicity via chitin synthase *MoCHS1*

Building on the METan module’s association with virulence, we prioritized its 55 candidate genes for validation of possible functions. Hub gene selection (module membership > 0.9, gene significance > 0.65) identified 20 high-confidence virulence effectors, 12 of which were significantly downregulated in both Δ*Motem1* and *MoTem1-CA* compared to WT (Fig. S10).

**Fig. 6:**
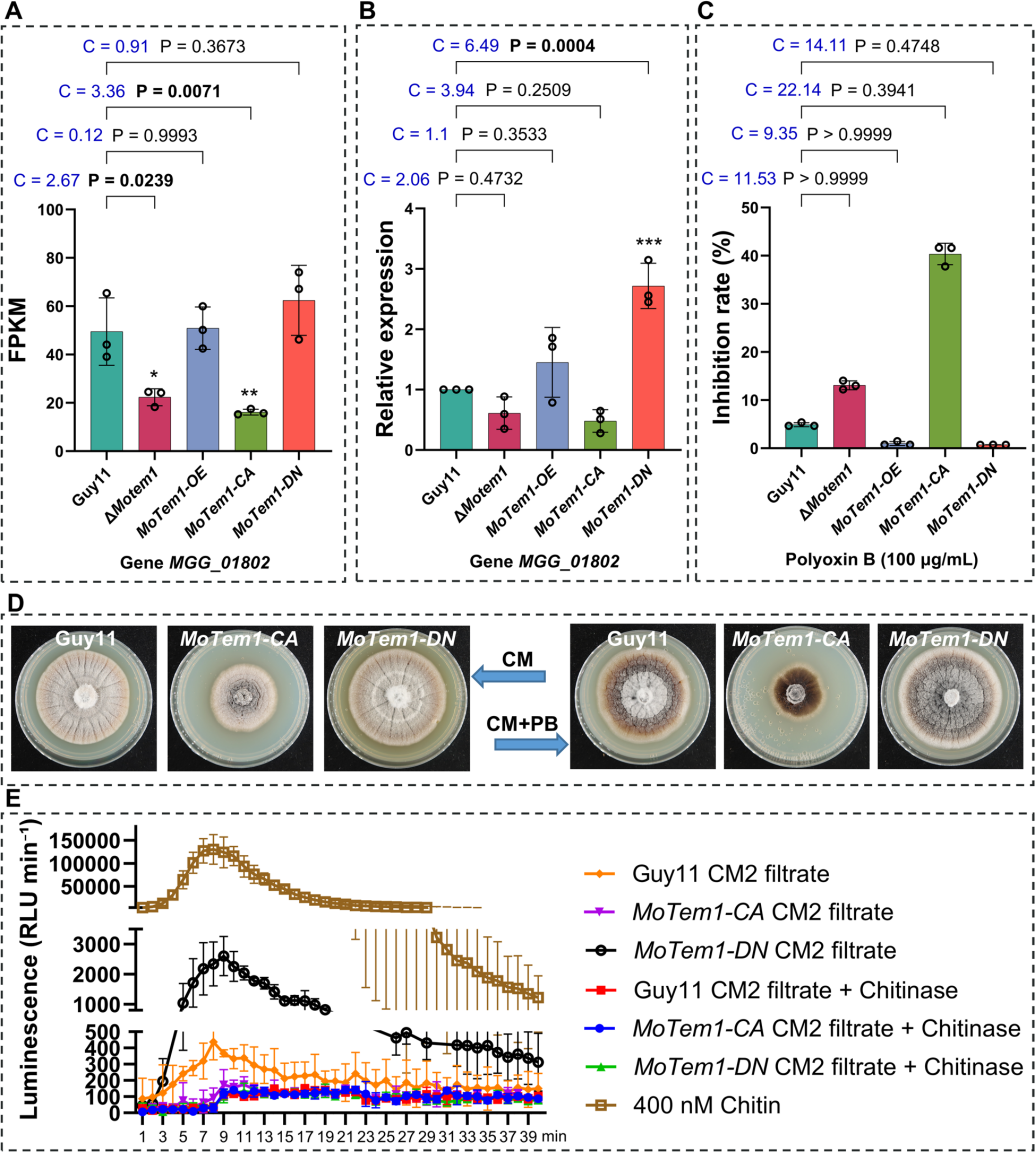
The chitinase gene MGG_01802 links MoTem1 signaling to chitin metabolism and pathogenicity. **A** FPKM values of MGG_01802 from transcriptomic data. Statistical significance was determined by one-way ANOVA with Dunnett’s post hoc test. data represent means ± SD, *n* = 3 biological replicates. **B** qRT-PCR analysis of MGG_01802 expression in different strains. Statistical significance was determined by one-way ANOVA with Dunnett’s post hoc test. data represent means ± SD, *n* = 3 biological replicates. **C** Inhibition rate of Polyoxin B (PB) (100 μg/mL) on various strains. Statistical significance was determined by Kruskal–Wallis test with Dunn’s post hoc test. data represent means ± SD, *n* = 3 biological replicates. For A, B and C, Statistical significance compared to Guy11 is indicated as follows: *p < 0.05, **p < 0.01, ***p < 0.001, and ****p < 0.0001. Cohen’s d (C) is reported as a measure of effect size, with thresholds defined as: 0.2 = small, 0.5 = medium, and 0.8 = large. **D** Colony morphology of Guy11, *MoTem1-CA*, and *MoTem1-DN* strains with or without PB (100 μg/mL). **E** ROS burst assay on rice leaf disks induced by sterile culture filtrates from indicated strains. Using chitohexaose (a chitin oligomer) at a final concentration of 400 nM as a positive control. Pretreatment with chitinase to eliminate chitin components in the culture filtrates abolished ROS induction, confirming that the response was specifically elicited by chitin oligomers

Among these, *MoCHS1* (MGG_01802), fifth from the top in Fig. S10, encoding a class V chitin synthase critical for cell wall integrity, exhibited expression patterns mirroring the virulence phenotypes: 3.07-fold downregulation in *MoTem1-CA* and 1.26-fold upregulation in *MoTem1-DN* versus WT (Fig. 6A and B). This transcriptional coupling suggests that MoTem1 activity at least indirectly affects *MoCHS1* expression. This hypothesis is further corroborated by pharmacological inhibition using Polyoxin B (100 μg/mL), a chitin synthase inhibitor. It suppressed *MoTem1-CA* growth by 40.36 ± 6.42% versus 4.89 ± 1.29% in WT, while *MoTem1-DN* remained resistant (Fig. 6C, D and Fig. S15).

To further test this hypothesis, we performed pharmacological inhibition using the chitin synthase inhibitor Polyoxin B (100 µg/mL) (Fig. 6C, 6D and Fig. S15). Compared to the wild type (WT; inhibition rate of 4.89 ± 0.42%), the *MoTem1-CA* exhibited extreme hypersensitivity, with an inhibition rate of 40.36 ± 2.23%. In stark contrast, *MoTem1-DN* showed strong resistance, with an inhibition rate of only 0.73 ± 0.01%. Although these differences were not statistically significant, the effect sizes were exceptionally large (Cohen’s d = 22.14 for *MoTem1-CA* and 14.11 for *MoTem1-DN*). These results underscore that MoTem1’ s GTP-bound state critically regulates cell wall integrity, with constitutive activation conferring severe drug hypersensitivity and a locked inactive state strongly promoting tolerance.

Since chitin oligomers, chitohexaose, is a standard elicitor of plant innate immunity reaction with ROS generation, we performed a standard ROS assay using culture filtrate from CM2 grown Guy11, *MoTem-CA*, and *MoTem-DN* strains without and with chitinase added. MoTem-DN elicited a much higher ROS response than Guy11 filtrates and especially *MoTem-CA* filtrates. The addition of chitinase abolished all responses, indicating that the presence of varying amounts of chitin oligomer-PAMP is mainly responsible for the plant ROS reaction (Fig. 6E). Additionally, ROS assays were performed for KO and OE strains, but the results showed no difference from Guy11, so not shown. These data suggest MoTem1 activity states may affect chitin biosynthesis. Constitutive activation in the mutant *MoTem1-CA* (Q182L) lowers *MoCHS1* expression and attenuates virulence, whereas the dominant-negative inhibition in the mutant *MoTem1-DN* (T137N) is connected with an elevated *MoCHS1* expression and a higher pathogenicity response resulting in larger lesions in the necrotrophic stage (Yang et al. 2019).

## Discussion

As a core component of the mitotic exit network (MEN), the small GTPase Tem1 is indispensable for cytokinesis in *S. cerevisiae*, where it coordinates spindle positioning with cell cycle progression (Scarfone and Piatti 2015). It has similar roles in *F. graminearum* (Miao et al. 2023). In this study, we identified MoTem1 as the functional homolog of yeast and *F. graminearum* Tem1 in *M. oryzae* and elucidated its roles in mitotic fidelity, stress tolerance, and pathogenicity. Our findings demonstrate that MoTem1 affects nuclear segregation, vegetative growth, stress resilience, as well as virulence, acting as an upstream regulatory nexus linking cell cycle regulation and growth to interactions with the biotic and abiotic environment in a way that is reminiscent of MoIsw2 regulation (Pei et al. 2024).

### Evolutionary conservation and divergence in Tem1 localization

The small GTPase Tem1 exhibits conserved spindle pole body (SPB) localization across fungi, including *S. cerevisiae*, *S. pombe*, and *F. graminearum*, where it regulates cell cycle progression (Sohrmann et al. 1998; Valerio-Santiago and Monje-Casas 2011; Milne et al. 2014; Fukada and Kubo 2015; Miao et al. 2023). Moreover, only the activated Tem1 can be located in SPB, or in other words, SPB only recruits Tem1 that is in an activated state (Zhou et al. 2024). In *M. oryzae*, MoTem1 similarly localizes to SPBs in hyphae and conidia, with its active state MoTem1^CA^ showing enhanced SPB enrichment and the inactive state MoTem1^DN^ displaying diffuse cytoplasmic distribution (Fig. 4E). This GTP-dependent SPB recruitment mirrors *S. pombe* Spg1 dynamics (Schmidt et al. 1997) but diverges from *F. graminearum*, where inactive FgTem1 localizes to septa (Miao et al. 2023). Note that inactive MoTem1 strains lack targeting to septa, as do inactive AgTem1 strains of *Ashbya gossypii* (Finlayson et al. 2011), suggesting lineage-specific adaptations in the MEN regulation. These findings highlight Tem1’s conserved role in mitotic control while underscoring species-specific localization mechanisms that may reflect different roles in growth and infection, underscoring also a looser connection between nuclear division and separation into different hyphal and conidial compartments in filamentous fungi than is the case for yeast mother and daughter cell separation.

### SPOC enforcement and GAP complex functionality in MEN regulation

The spindle position checkpoint (SPOC) safeguards mitotic fidelity by delaying MEN activation until spindle alignment is verified (Falk et al. 2016). Central to this process is the Bfa1-Bub2 GAP complex, which hydrolyzes Tem1-bound GTP to enforce mitotic arrest (Scarfone and Piatti 2015). In *M. oryzae*, MoBfa1 interacts with both active and inactive MoTem1, while MoBub2 binds exclusively to the GTP-bound form (Fig. 1B, C, D). This mirrors *S. cerevisiae*, where Bfa1 scaffolds Bub2-Tem1 interactions despite minimal GAP activity (Wang et al. 2000; Geymonat et al. 2002). We propose that MoBfa1 spatially confines MoBub2 near MoTem1, enabling rapid termination of the cell cycle signalling post-cytokinesis. Intriguingly, yeast ScBfa1’s N-terminal domain regulates SPOC independently of GAP activity by blocking Tem1-SPB association (Li and Song 2022). Analogously, MoBfa1’s persistent interaction with inactive MoTem1 suggests a dual role: tethering Bub2 for MEN regulation and enforcing SPOC through structural motifs. These mechanisms collectively ensure precise coordination between spindle positioning and mitotic exit, preventing aneuploidy in filamentous fungi.

### Nuclear segregation defects and implications for pathogenicity

Nuclear segregation through cytokinesis, a hallmark of cell division, is tightly regulated by the MEN pathway via Tem1 (Walther and Wendland 2003). In *C. albicans*, CaTem1 deletion mutants exhibit multinucleate cells due to defective cytokinesis (Milne et al. 2014). Similarly, Δ*Motem1* displayed hyphal compartments harboring up to four nuclei, indicating severe nuclear segregation defects compared to Guy11 (WT). Strikingly, the constitutively active *MoTem1-CA* strain also showed multinucleate phenotypes (Fig. 4E), likely due to premature MEN activation bypassing the spindle position checkpoint (SPOC) surveillance. This is analogous to a mechanism in Bub2 deletion mutants that results in multinucleate cells, because Tem1 hyperactivity disrupts mitotic exit timing (Geymonat et al. 2002; Fukada et al. 2019). Paradoxically, the dominant-negative *MoTem1-DN* strain maintained normal nuclear counts despite MEN pathway inactivation (Fig. 4E). However, this mirrors observations in *F. graminearum*, where introducing inactive FgTem1^T118N^ rescued septation defects in Δ*Fgbub2* mutants (Miao et al. 2023). We thus propose that inactive MoTem1 recruits compensatory factors during septation, circumventing fungal compartment ploidy abnormalities caused by MEN dysregulation (Fig. 4E). These findings collectively implicate SPOC-MEN crosstalk in ensuring nuclear fidelity with Tem1 activity states in control of septation.

### Effects on pathogenicity by MoTem1 activity states and chitin synthase modulation

Δ*Motem1* deletion strains showed modest pathogenicity reduction when it comes to the number of lesions, similar to what was found for *F. graminearum* Δ*Fgtem1* with conidia retaining normal appressorium formation (Miao et al. 2023). On the other hand, deletion harmed the growth of the lesion size, which is dependent on the biotrophic growth of the fungus between adjacent leaf cells through plasmodesmata (Kankanala et al. 2007; Fernandez and Orth 2018). Strikingly but fitting well with the growth rate (Fig. 2B), *MoTem1-DN* exhibited hypervirulence, likely via MEN pathway inactivation, mirroring *C. albicans* virulence regulation by CaCdc5 (Bachewich et al. 2003; Scarfone and Piatti 2015). The effect of this is likely to promote hyphal growth, as for inactivation of *C. albicans* Tem1 (Milne et al. 2014). In *M. oryzae,* such inactivation promoting invasive hyphae growth should result in increased lesion size as lesion expansion is the known pathogenicity characteristic that is directly dependent on the extent of invasive hyphal growth (Zhang et al. 2024), and that is also what we observe as *MoTem1-DN* shows larger lesions than Guy11 (Fig. 3A).

On the contrary, *MoTem1-CA* impaired growth, sporulation, and virulence due to unchecked MEN signaling overriding SPOC controls, as also seen in *C. orbiculare*, *C. higginsianum*, and *M. oryzae* Δ*Mobub2* mutants (Fukada and Kubo 2015; Fukada et al. 2019).

Transcriptomics revealed a most probably downstream indirect effect of MoTem1 states on the regulation of *MoCHS1*, as *MoTem1-CA* negatively affects *MoCHS1* expression while *MoTem1-DN* has positive effects, creating hypervirulence (Fig. 6A and B). *M. oryzae* has seven identified chitin synthases, and *MoCHS1* (a Class V chitin synthase) is one of the three needed for pathogenicity (Kong et al. 2012). Conidial spore tip mucilage, important for sticking the conidium to leaf surfaces were absent in *MoCHS1* mutants (Kong et al. 2012). Thus, an increase of such mucilage necessary for infection (Hamer et al. 1988) in *MoTem1-DN* this might be part of the reason for the increased pathogenicity of *MoTem1-DN* giving the infection a faster start promoting larger lesions (Fig. 3A) Inhibition of CHS with Polyoxin B corroborated the positive effect of *MoTem1-DN* on *MoCHS1* expression as the colonial growth rate, with *MoTem1-CA* showed 40.36 ± 6.42% growth inhibition versus 4.89 ± 1.29% in Guy11 (WT), while *MoTem1-DN* was resistant (Fig. 6C). Inhibition of CHS with Polyoxin B corroborated the positive effect of *MoTem1-DN* on *MoCHS1* expression, as the colonial growth rate, with *MoTem1-CA* showed 40.36 ± 6.42% growth inhibition versus 4.89 ± 1.29% in Guy11 (WT), while *MoTem1-DN* was resistant (Fig. 6C and D). The differences in MoCHS expression were clearly reflected by plant immunity responses, showing the highest response for *MoTem1-DN* followed by Guy11, and thereafter *MoTem1-CA.* A high and imbalanced MoChs1 expression might release more chitin oligomers or more expose the fungal cell wall chitin to plant extracellular (apoplastic) endochitinases (Sundin et al. 1991), generating the chitooligomers that is percieved as PAMPs by the plant and trigger an early entry in the necrotrophic stage with formation of larger necroses.

**Fig. 7:**
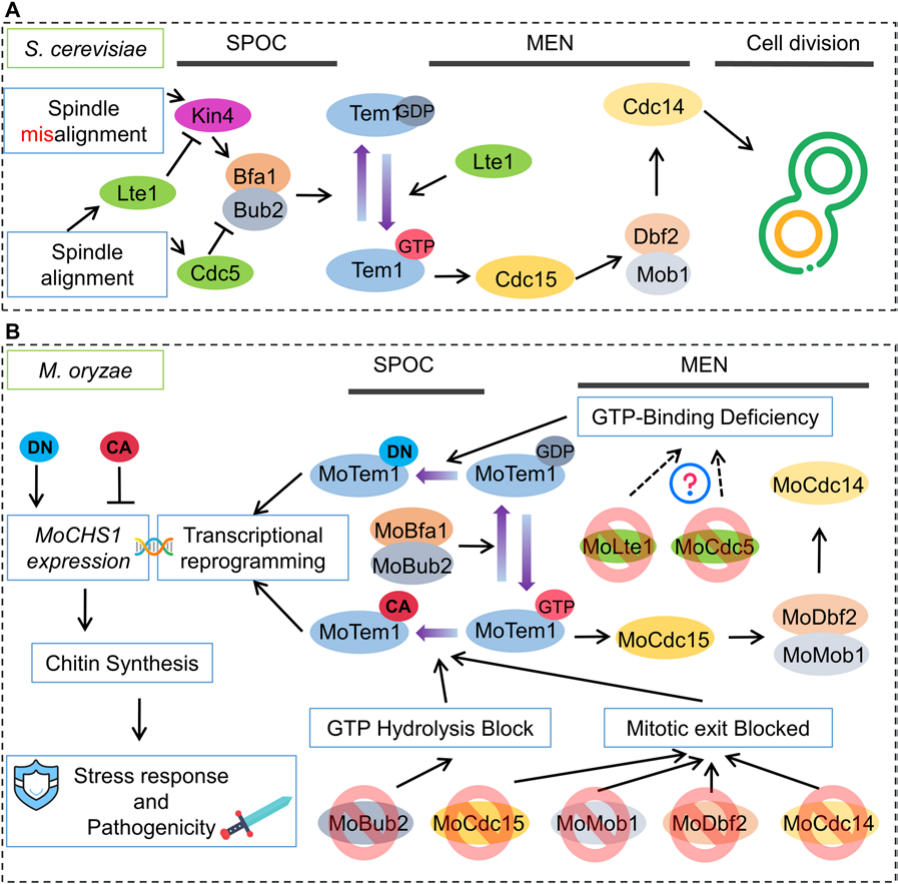
Connecting MoTem1-mediated mitotic exit to stress adaptation and pathogenicity. **A** Summarizes the known signaling pathway of MEN-related proteins in yeast from SOPC to cell division. When the spindle arrangement is incorrect, Kin4 activates Tem1 through Bfa1, transitioning it from an activated state to an inactive state, thereby keeping MEN in a closed state. Conversely, when the spindle arrangement is correct, Lte1 inhibits Kin4 and cdc5 inhibits Bub2, rendering the GAP complex ineffective. Simultaneously, Lte1 promotes the transition of Tem1 from an inactive state to an activated state. The activated Tem1 initiates sequential signal transmission to the Cdc15, Dbf2-Mob1 complex, and Cdc14, ultimately completing the initiation of MEN and cell division. **B** Summarizes the correlation between MEN-related proteins in *M. oryz*ae and stress responses, as well as pathogenicity. First, the deletion of MoBub2 prevents MoTem1 from activating GTPase activity, thereby blocking its transition from the activated state to the inactive state, resulting in a persistently activated state (CA). Subsequently, the MoTem1 deletions have effects downstream of MoTem1. MoCdc15\\ MoMob1\\ MoDbf2\\ MoCdc14 are all affected, leading to impaired mitotic exit and lack of feedback, maintaining MoTem1 in a persistently activated state (CA). We hypothesize that deletions of MoLte1 or MoCdc5 would result in insufficient GTP binding (insufficient GFF, while GAP activity remains uninhibited), leading to a persistently inactive state (DN). These persistently activated and inactive states induce transcriptional reprogramming in the rice blast pathogen *M. oryzae*. Specifically, MoTem1's CA inhibits the gene expression of MoCHS1, whereas DN promotes it, and MoCHS1 expression is positively correlated with stress responses and pathogenicity

We summarized the known reactions reactions of Tem1 yeast and other organisms (Scarfone and Piatti 2015; Falk et al. 2016; Ibrahim 2025) and compared that with what we have now found for M. oryzae in a simple models of the signalling pathway (Fig. 7A and B) In short, the activity forms of MoTem1 (CA or DN) have profound effects on transcription that have effects on stress tolerance abut also on pathogenicity. One of the main effects is on pathogenicity, most likely through the regulation of the MoChs1 chitin synthase (Fig. 6), important for the fungal cell wall formation and release of chitin as a well-known PAMP, with a strong effect on plant innate immunity responses and with that plant ROS formation (Fig. 6E).

## Conclusion

In summary, this study establishes MoTem1 as a global upstream regulator of the mitotic exit network (MEN) in *M. oryzae*, with its functional states orchestrating direct and indirect divergent cellular outcomes most likely in collaboration with upstream global regulators like MoIsw2 that have epigenetic regulatory effects balancing growth with biotic and abiotic interactions (Pei et al. 2024). We demonstrate that the GTP-bound (active) state of MoTem1 drives MEN pathway activation, ensuring precise cytokinesis and nuclear segregation into hyphal compartments by hyphal septation and has many downstream direct and indirect regulatory effects with importance for growth, stress tolerance, and pathogenesis.

## Materials and methods

### Gene identification and phylogenetic tree

The *M. oryzae* Tem1 homolog (MoTem1, MGG_04862) was identified via BLASTp homology search using *S. cerevisiae* Tem1 (ScTem1, NP_013647.1) and *F. graminearum* FgTem1 (Miao et al. 2023) as queries against the *M. oryzae* 70–15 genome (NCBI taxid: 242,507). To resolve evolutionary relationships, Tem1 homologs from 22 fungal taxa (21 pathogens) were retrieved and aligned using MUSCLE. A maximum-likelihood phylogenetic tree was constructed in MEGA v11.0 (Tamura, Stecher and Kumar, 2021). Accession numbers and species are listed in Table S1.

### Strain construction

Targeted gene deletion and complementation were performed as described (Chen et al. 2023). Generation *ΔMotem1*: A ~ 800 bp 5’ and 3’ flanking regions were amplified and fused to a hygromycin resistance cassette (HYG), followed by polyethylene glycol (PEG)-mediated protoplast transformation. Primary transformants were selected on hygromycin (200 μg/mL) and validated by Southern blot. Complementation constructs (1.5 kb native promoter + GFP + *MoTEM1* or mutant alleles) were cloned into pKNT and reintroduced into Δ*Motem1*. GFP fluorescence was confirmed via confocal microscopy (Nikon A1, Japan).

For overexpression (OE), constitutively active (CA; Q182L), and dominant-negative (DN; T137N) alleles, MoTem1 variants were cloned into pKNT-RP27 (Chen et al. 2008) and transformed into Guy11. The strains expressing MoTEM1, *MoTEM1-Q182L,* or *MoTEM1-T137N* were verified by RT-qPCR. Primer sequences are provided in Table S2.

### Culture conditions and other strains used

The *M. oryzae* wildtype strain Guy11 served as the background for generating the following mutants: Δ*Motem1* (gene deletion), *MoTem1-OE* (overexpression), *MoTem1-CA* (constitutively active allele), and *MoTem1-DN* (dominant-negative allele). The complementation strains *MoTem1-OE*, *MoTem1-CA,* and *MoTem1-DN* were derived from the Δ*Motem1* background. Unless specified, fungal strains were maintained on a complete medium (CM II) at 26 °C under dark conditions (40% relative humidity). Escherichia coli DH5α was used for routine plasmid propagation, BL21(DE3) for recombinant protein expression, and *S. cerevisiae* AH109 for yeast two-hybrid assays. Media compositions and applications are detailed in Table S3.

### Southern blot assay and reverse transcript quantitative PCR

Southern blotting followed Biregeya’s work (Biregeya et al. 2022). Genomic DNA was digested with Hind III (Thermo Fisher), electrophoresed on 0.8% agarose, and transferred to Hybond-N + membranes (GE Healthcare). A DIG-labeled probe targeting *MoTEM1* was hybridized using the Roche DIG High Prime DNA Labeling and Detection Kit.

For RT-qPCR, total RNA was extracted with the Promega Total RNA Kit (LS1040) and reverse-transcribed using Evo M-MLV RT Premix (AG11728). The following qPCR step amplification and detection utilized SYBR Green PRO Taq HS Premix (AG11701) on a Bio-Rad CFX96 system. *MoACTIN* (MGG_03982) served as the endogenous reference gene with stable expression (Anjago et al. 2022). Relative expression was calculated via the 2^−ΔΔCT^ method (Livak and Schmittgen 2001).

### Yeast two-hybrid (Y2H) and pull-down assays

Y2H interactions were performed using the Matchmaker Gold System (Clontech). MoTem1-Q182L (bait) and MoTem1-T137N (bait) were cloned into pGBKT7 (BD), while putative interactors (MoBfa1 [MGG_03162], MoBub2 [MGG_04676]) were cloned into pGADT7 (AD). Co-transformed AH109 cells were plated on SD/-Leu/-Trp and SD/-Ade/-His/-Leu/-Trp agar media.

For pull-down assays, GST- or His-tagged proteins were expressed in BL21(DE3) (0.1 mM IPTG, 16 °C) and affinity-purified using glutathione-agarose beads (Smart-lifesciences). Binding complexes were resolved by SDS-PAGE and immunoblotted with anti-GST (1:5,000) and anti-His (1:3,000) antibodies (Takara Bio). Signals were detected using Advansta WesternBright ECL and a Tanon 5200 imaging system.

### Growth and stress assessments

Radial growth was assessed on CM II (nutrient-rich) and minimal medium (MM; nutrient-limited). For stress assays, strains were inoculated on CM containing 10 mM H_2_O_2_, 0.1% Congo Red (CR), 0.01% SDS, 1 M sorbitol, 1 M NaCl, or 1 M KCl. Plates were incubated at 26 °C for 7 days, and relative inhibition rates were calculated as described previously (Biregeya et al. 2022). To further quantify the sensitivity of strains to each stress agent, dose–response assays were performed to determine the half-maximal inhibitory concentration (IC_50_). A gradient of concentrations was used for each stressor: for H_2_O_2_, 10, 5, 2.5, and 0 mM; for Congo Red (CR), 0.1%, 0.05%, 0.025%, and 0%; for SDS, 0.01%, 0.005%, 0.0025%, and 0%; and for NaCl, 1, 0.5, 0.25, and 0 M. After incubation at 26℃ for 7 days, the relative inhibition rate at each concentration was calculated as previously described. The resulting data (concentration versus normalized growth response) were then subjected to nonlinear regression analysis using GraphPad Prism 10 software, applying the "log[inhibitor] vs. normalized response-Variable slope" model, to calculate the IC_50_ value for each strain under each stress condition.

### Conidial germination and plant infection

Conidia were aseptically harvested from 10-day-old CM cultures by flooding with sterile water, the washings containing conidia adjusted to 1 × 10^4^ conidia/mL, and spotted on hydrophobic coverslips. Germination and appressorium formation were monitored at 2–24 h post-inoculation (hpi) using a Nikon Eclipse Ni microscope (Jiang et al. 2024).

For plant infections, 4-week-old rice seedlings (cv. CO39) were spray-inoculated with 3 × 10^4^ conidia/mL. Inoculated plants were maintained at 26 °C (> 90% humidity) for 5- 7 days. Disease severity was scored on a 5-grade scale based on lesion size: grade 1 (< 0.05 mm), grade 2 (0.05- 0.2 mm), grade 3 (0.2- 0.3 mm), grade 4 (0.3- 0.4 mm), and grade 5 (> 0.4 mm) (Chen et al. 2023).

### GTPase enzyme activity assay

GTPase enzyme activity was determined using a one-step sandwich enzyme-linked immunosorbent assay (ELISA) with the Microorganism GTPase ELISA Kit (REF: YJ921558). To differentiate its function under various conditions, the enzymatic activity was measured separately using purified proteins in vitro and within total proteins extracted from mycelia in vivo.For the in vitro GTPase activity assay, 5 mg of purified GTPase protein was used as the test sample. For the in vivo GTPase activity assay, 1 mg of total protein extracted from mycelia served as the sample. All protein samples were diluted to appropriate concentrations using the recommended buffer.

The specific detection procedure was as follows: The prepared samples and a series of standard solutions at known concentrations were added separately to the wells of a microplate pre-coated with a GTPase-specific antibody. Subsequently, horseradish peroxidase (HRP)-conjugated detection antibody was added. After incubation and thorough washing, the substrate 3,3',5,5'-tetramethylbenzidine (TMB) was added for color development. TMB turns blue catalyzed by peroxidase and finally changes to yellow upon the addition of an acidic stop solution. The intensity of the color is positively correlated with GTPase activity in the sample. The absorbance (OD value) of each well was measured at a wavelength of 450 nm using a microplate reader. The GTPase activity units in the samples were calculated by comparing the OD values against the standard curve..

### Confocal microscopy observations

Subcellular localization was analyzed using a Nikon A1 confocal microscope. EGFP/mCherry-tagged strains were cultured on CM II, fixed in 4% paraformaldehyde, and imaged with a 60 × oil immersion lens. Z-stacks (0.5 μm steps) were acquired using blue light excitation (EGFP) (argon laser 488, excitation filter:470/40, dichromatic mirror: 495LP, barrier filter: 515/30, relative brightness EGFP vs EGFP 100%), or green light excitation (mCherry) (He–Ne laser 543, excitation filter:560/55, dichromatic mirror: 590LP, Barrier filter: 630/60 (light imaged)**,** relative brightness of mCherry vs EGFP 47%). Live-cell imaging of hyphae was conducted on CM II agar pads at 26 °C, capturing time-lapse sequences at 2-min intervals. Images were deconvolved (NIS-Elements v5.21) and analyzed for fluorescence intensity and colocalization.

### Transcriptome analysis

RNA from Guy11 (Wildtype), Δ*Motem1*, *MoTem1-CA*, *MoTem1-DN,* and *MoTem1-OE* strains (triplicates) was extracted with TRIzol, and further sequenced (Illumina NovaSeq 6000, 150 bp PE) by Qingke Biotechnology and aligned to the *M. oryzae* 70–15 genome (HISAT2). Differential expression (DESeq2; FDR < 0.01, |log2FC|> 2) and functional enrichment (KEGG/GO) were analyzed.

### Plant innate immunity activity analysis of chitin-derived elicitors in culture filtrates

The activity of chitin-derived elicitors for plant innate immunity reactions released by *M. oryzae* strains was assayed. Wild-type and mutant strains were first cultured on CM2 plates for 7 days. Mycelial plugs of equal area were excised, fragmented, and inoculated into liquid CM2 medium for shake culture. When approximately 70–80% of the mycelial pellets reached a diameter of 2–3 mm, the culture broth was harvested. The broth was filtered through a 0.22 μm membrane to obtain sterile cell-free supernatant (CFS). For chitinase treatment, two equal aliquots of CFS (100 μL each) were used. One aliquot was supplemented with 20 μL of a chitinase stock solution (Solarbio, Cat# C9331; 10 mg/mL in 50 mM sodium acetate buffer, pH 5.5), while the other received an equal volume of buffer as an untreated control, bringing the final volume of each reaction to 120 μL. The chitinase-supplemented sample was incubated at 37℃ in the dark for 2 h, followed by heat inactivation at 95 °C for 10 min. The control sample was incubated identically but without the heat-inactivation step. All samples were then centrifuged at 14,000 × g for 15 min at 4 °C, and the supernatants were collected for subsequent reactive oxygen species (ROS) burst assays.

ROS production was measured using a chemiluminescence-based method. Rice leaf discs, pre-incubated overnight in the dark, were placed in a white 96-well plate. Each well was loaded with 50 μL of detection solution containing L-012 (Chemiluminescent ROS probe) (Fujifilm, Cat# LCK-012; 20 μM) and horseradish peroxidase (Macklin, Cat# P8125; 2.5 μg/mL). After a 30-min equilibration in the dark, 50 μL of the test sample (or 800 nM chitohexaose solution as a positive control, yielding a final well concentration of 400 nM) was added. Luminescence was immediately recorded every 1 min for 40 min using a chemiluminescence microplate reader.

### Statistical analysis

The statistical analysis in this study employed a rigorous workflow to ensure test appropriateness. Normality was assessed within each group using the Shapiro–Wilk test. For data meeting the normality assumption, the homogeneity of variances was evaluated via the Brown-Forsythe test. If both assumptions were satisfied, parametric tests were selected: an independent samples t-test for two-group comparisons, or a one-way ANOVA with Dunnett’ s post hoc test for comparisons involving three or more groups. If either the normality or homogeneity of variances assumption was violated, non-parametric tests were applied: the Mann–Whitney U test for two-group comparisons, or the Kruskal–Wallis test with Dunn’ s post hoc test for multiple comparisons. The significance level for all analyses was set at α = 0.05.

## Supplementary Information

Supplementary Material 1: Fig. S1. Construction verification, gene expression of Moem1 mutant strains, and MoTem1 localization. (A) Schematic diagrams of MoTem1 and ScTem1. The bar represents 30 amino acids. Schematic representation of activating and inactivating mutations. (B) Prediction of MoTem1's activation and inactivation sites. The Accession numbers of ScTem1, FgTem1, CoTem1 and MoTem1 were NP_013647.1, XP_011328835.1, TDZ19252.1 and XP_003712342.1, respectively. (C) Southern blot analysis of ΔMotem1. In the wild-type genome, Hind III digestion produced a 2040 bp fragment, whereas in the ΔMotem1 genome, Hind III digestion produced a 4035 bp fragment. Bar represents 1 kb. (D) Validation of MoTem1 expression in overexpression and mutant strains. EGFP fluorescence was first observed to confirm protein expression in the respective strains. Subsequently, RT-qPCR analysis was performed to quantify MoTem1 transcript levels, using MoACTIN (MGG_03982) as the internal reference. Statistical significance compared to Guy11 is indicated as ****p < 0.0001 (one-way ANOVA with Dunnett’s post hoc test; data are means ± SD, *n* = 4 biological replicates).

Supplementary Material 2: Fig. S2. Conidia formation and conidia germination. (A) The process of conidial germination in different strains. Scale bar, 10 μm. (B) The germination rates of conidia at various time points for different strains. Statistical significance was determined by one-way ANOVA with Dunnett’s post hoc test. data represent means ± SD, n = 3 biological replicates. Statistical significance compared to Guy11 is indicated as follows: *p < 0.05, **p < 0.01, ***p < 0.001, and ****p < 0.0001. Cohen’s d (C) is reported as a measure of effect size, with thresholds defined as: 0.2 = small, 0.5 = medium, and 0.8 = large.

Supplementary Material 3: Fig. S3. Rice leaf lesion caused by infection from conidia of different strains. Conidial suspensions (1×105 conidia/mL in 0.02% Tween 20) were sprayed onto the rice leaves, and the disease symptoms were analyzed at 5 dpi.

Supplementary Material 4: Fig. S4. Colony morphology in response to different strains to various stress treatments on CM-medium using one standard concentration of stressing agent. H2O2 represents oxidative stress, CR and SDS represent cell wall stress, and NaCl, KCl, and Sorbitol represent osmotic stress.

Supplementary Material 5: Fig. S5 Inhibition rates and IC50 values for a range of different concentrations of stressing agents. The half-maximal inhibitory concentration (IC50) was determined to quantify strain sensitivity. Strains were exposed to gradients of H2O2, Congo Red (CR), SDS, and NaCl. After incubation, inhibition rates were calculated. IC50 values were derived by nonlinear regression (GraphPad Prism 10, log[inhibitor] vs. normalized response-Variable slope model).

Supplementary Material 6: Fig. S6. Transcriptomic analysis overview. (A) Presents a box plot of transcriptomic data, demonstrating highly consistent median expression levels and distribution ranges across groups, indicating no significant batch-related bias. (B) Shows a correlation heatmap of transcriptomic data, with correlation analysis indicating strong biological reproducibility. (C) A PCA plot, revealing that samples cluster primarily by experimental group, where the OE group and the GUY11 control group exhibit high transcriptional similarity, consistent with phenotypic observations.

Supplementary Material 7: Fig. S7 Analysis of differentially expressed genes. (A) Shows a differential gene count chart with a similar function as a Venn diagram, showing 72 shared differentially expressed genes (DEGs) compared to Guy 11 (WT). (B-E) Displays GO classification analysis for the DEGs of the four strains (⊿ Motem1, MoTem1-OE, MoTem1-CA, and MoTem1-DN).

Supplementary Material 8: Fig. S8 Weighted gene co-expression network analysis (WGCNA). Eight distinct modules, named by different colors, were identified.

Supplementary Material 9: Fig. S9 Module-trait relationships found by the weighted gene co-expression network analysis (WGCNA). Note especially that the Metan module is strongly correlated with growth rate and pathogenicity.

Supplementary Material 10: Fig S10 the FPKM values of the top 20 genes in MeTan for KO and MoTem1-CA strains. Note especially that the chitin synthase MoCHS1 (MGG_01802) is among the genes characterizing the difference between Guy11, △ Motem1, and MoTem1-CA, contributing to the WGCNA identification of the MeTan module strongly correlated with growth rate and pathogenicity.

Supplementary Material 11: Fig S11 RT-qPCR validation of RNAsec values for some of the 72 DEGs common to all strains. (A) Three selected genes for MeBrown correlate well. (B) Three selected genes for MeTan correlate well. The MoChs1 (MGG_01802) is one of the genes.

Supplementary Material 12: Fig. S12. Association between the MeBrown module and growth rate. The heatmap shows gene expression in different strains, where green indicates low expression and red indicates high expression, illustrating gene expression profile differences among the samples.

Supplementary Material 13: Fig. S13. Association between the MeYellow module and growth rate. The heatmap shows gene expression in different strains, where green indicates low expression and red indicates high expression, illustrating gene expression profile differences among the samples.

Supplementary Material 14: Fig. S14 Association between the MeTan module and pathogenicity. The heatmap shows gene expression in different strains, where green indicates low expression and red indicates high expression, illustrating gene expression profile differences among the samples.

Supplementary Material 15: Fig S15 Colony phenotypes on CM and on CM + polyoxin B. Polyoxin B is a chitin synthase inhibitor, with a working concentration of 100 μg/mL.

Supplementary Material 16: Supplementary Table 1. Culture Media and Applications. Supplementary Table 2. Primer Sequence and Applications. Supplementary Table 3. Accession number of tem1 homologs in different species. Supplementary Table 4. GO enrichment analysis of differentially expressed genes in various strains. Supplementary Table 5. Data plotting and statistical analysis methodology.

## Data Availability

All fungal strains and plasmids generated in this study are available from the corresponding authors upon reasonable request. The raw RNA-seq transcriptome data have been deposited in the NCBI Sequence Read Archive (SRA) under the BioProject accession number PRJNA1398772.

## Abbreviations

MEN: Mitotic exit network
TEM1: Termination of M-phase Protein 1
GAP: GTPase-activating protein
SPB: Spindle pole body
SPOC: Spindle position checkpoint
CDK: Cyclin-dependent kinase
DON: Deoxynivalenol
DEG: Differentially expressed gene
GO: Gene Ontology
KEGG: Kyoto Encyclopedia of Genes and Genomes
WGCNA: Weighted gene co-expression network analysis
*MoTEM1*: This represents the gene
MoTem1: This represents the protein
MoTem1^Q182L^: This represents MoTem1 after the Q182L mutation
MoTem1^T137N^: This represents MoTem1 after the T137N mutation
Δ*Motem1*: Knockout mutant of the MoTEM1 gene
Δ*Motem1*-Com: Complemented strain of the MoTEM1 knockout mutant
*MoTem1-OE*: Overexpression strain of MoTEM1
*MoTem1-CA*: Constitutively active strain of MoTEM1
*MoTem1-DN*: Dominant-negative strain of MoTEM1
