# Supplementary figures and images for: Mutational analysis of the mitotic exit GTPase MoTem1 reveals its role in development, stress adaptation, pathogenicity and global gene regulation in Magnaporthe oryzae
Source: Stress Biol. 2026 May 9;6(1):38. doi: 10.1007/s44154-026-00310-8 (PMC13157382; doi:10.1007/s44154-026-00310-8)

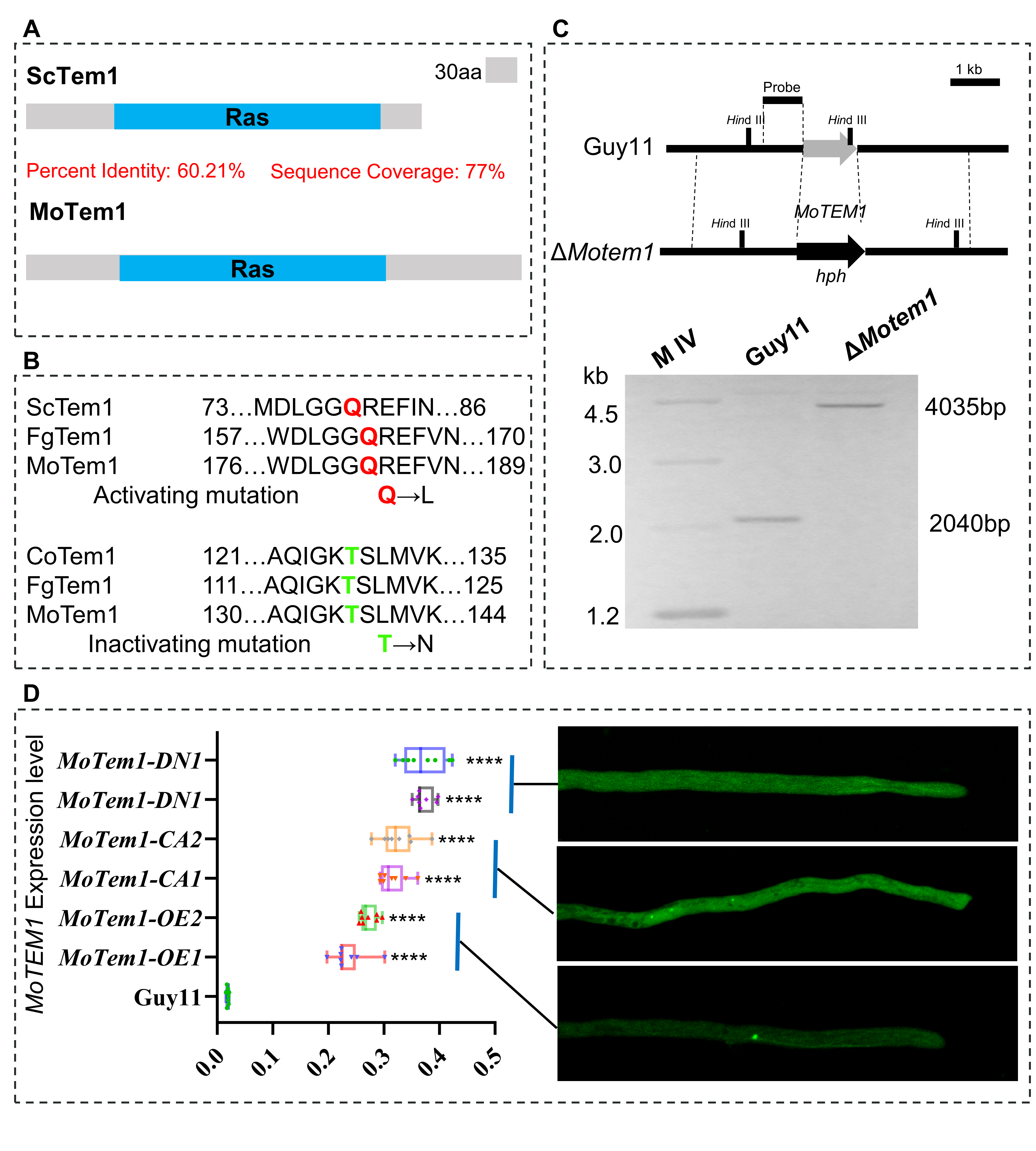

Supplement: Supplementary file 1 — Supplementary Material 1: Fig. S1. Construction verification, gene expression of Moem1 mutant strains, and MoTem1 localization. (A) Schematic diagrams of MoTem1 and ScTem1. The bar represents 30 amino acids. Schematic representation of activating and inactivating mutations. (B) Prediction of MoTem1's activation and inactivation sites. The Accession numbers of ScTem1, FgTem1, CoTem1 and MoTem1 were NP_013647.1, XP_011328835.1, TDZ19252.1 and XP_003712342.1, respectively. (C) Southern blot analysis of ΔMotem1. In the wild-type genome, Hind III digestion produced a 2040 bp fragment, whereas in the ΔMotem1 genome, Hind III digestion produced a 4035 bp fragment. Bar represents 1 kb. (D) Validation of MoTem1 expression in overexpression and mutant strains. EGFP fluorescence was first observed to confirm protein expression in the respective strains. Subsequently, RT-qPCR analysis was performed to quantify MoTem1 transcript levels, using MoACTIN (MGG_03982) as the internal reference. Statistical significance compared to Guy11 is indicated as ****p < 0.0001 (one-way ANOVA with Dunnett’s post hoc test; data are means ± SD, n = 4 biological replicates). [file 44154_2026_310_MOESM1_ESM.tif]

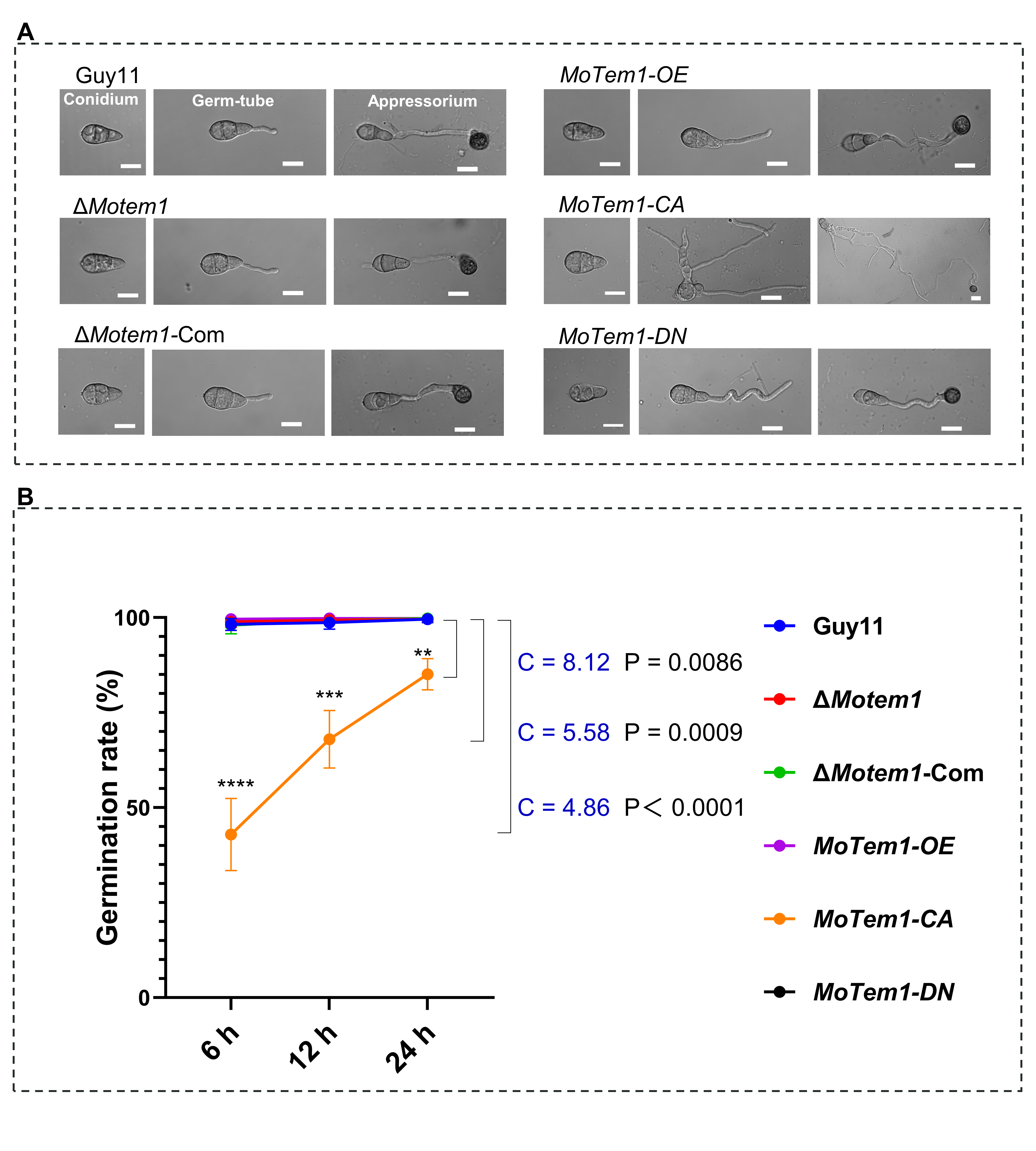

Supplement: Supplementary file 2 — Supplementary Material 2: Fig. S2. Conidia formation and conidia germination. (A) The process of conidial germination in different strains. Scale bar, 10 μm. (B) The germination rates of conidia at various time points for different strains. Statistical significance was determined by one-way ANOVA with Dunnett’s post hoc test. data represent means ± SD, n = 3 biological replicates. Statistical significance compared to Guy11 is indicated as follows: *p < 0.05, **p < 0.01, ***p < 0.001, and ****p < 0.0001. Cohen’s d (C) is reported as a measure of effect size, with thresholds defined as: 0.2 = small, 0.5 = medium, and 0.8 = large. [file 44154_2026_310_MOESM2_ESM.tif]

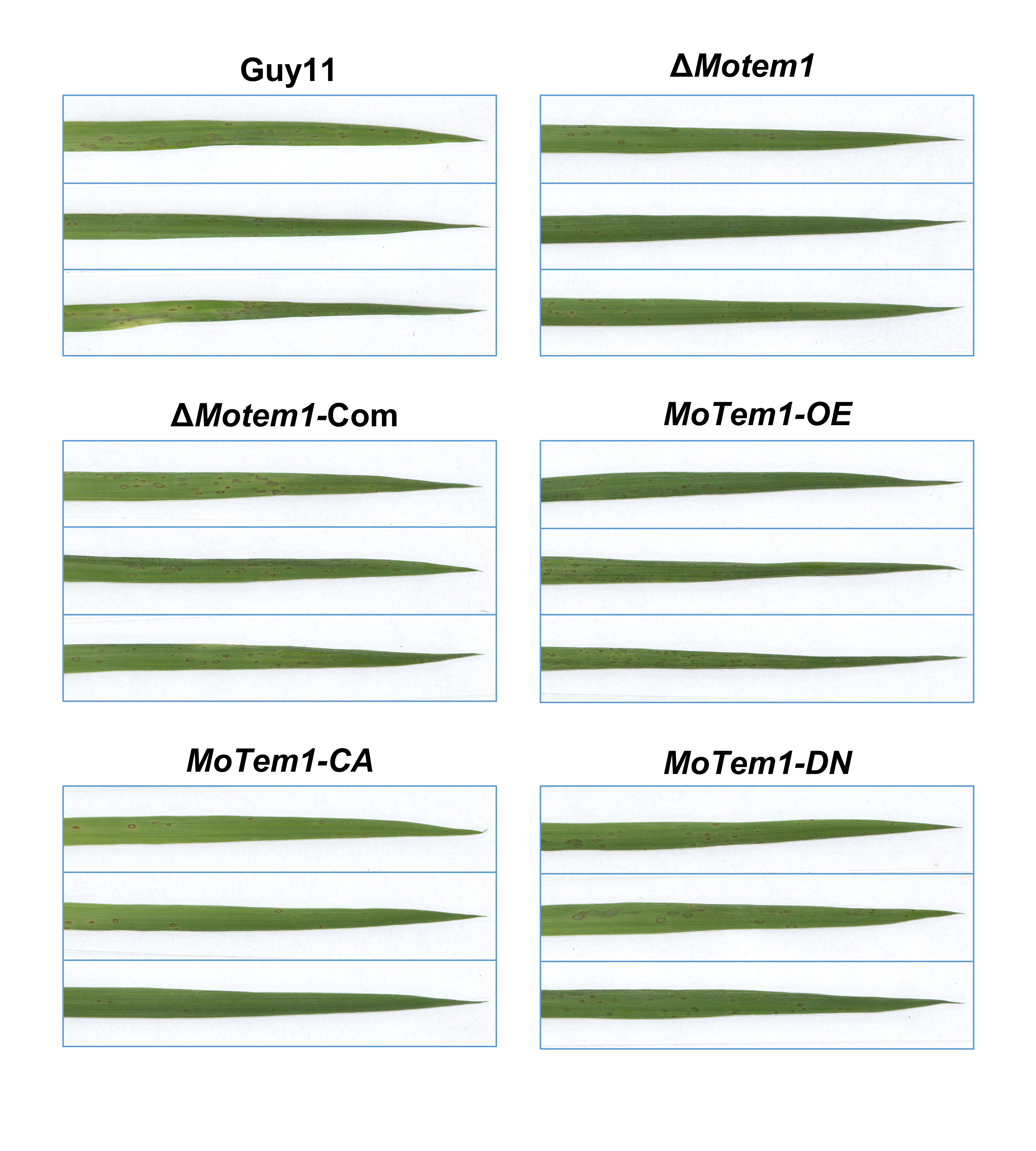

Supplement: Supplementary file 3 — Supplementary Material 3: Fig. S3. Rice leaf lesion caused by infection from conidia of different strains. Conidial suspensions (1×10^5^ conidia/mL in 0.02% Tween 20) were sprayed onto the rice leaves, and the disease symptoms were analyzed at 5 dpi. [file 44154_2026_310_MOESM3_ESM.tif]

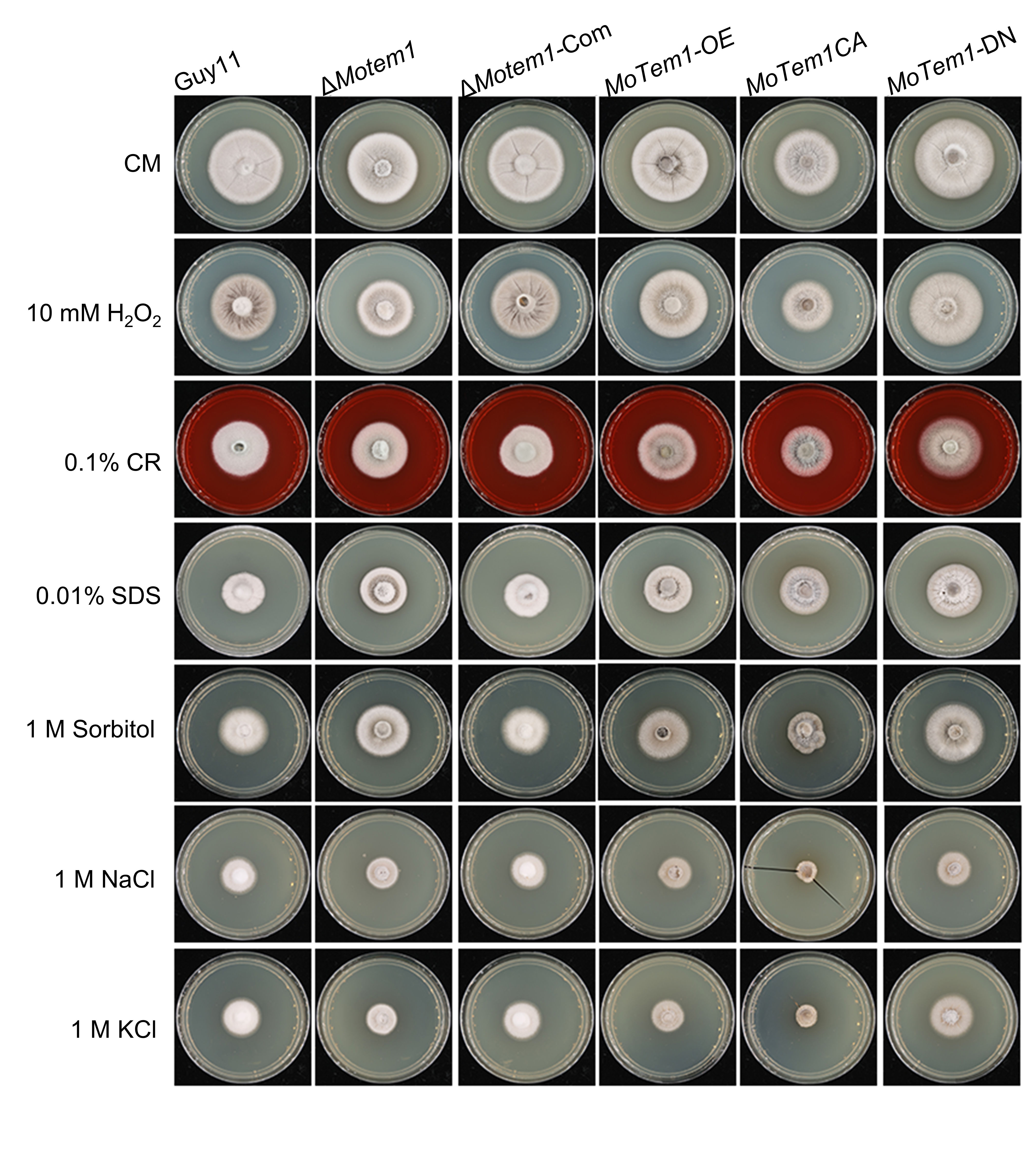

Supplement: Supplementary file 4 — Supplementary Material 4: Fig. S4. Colony morphology in response to different strains to various stress treatments on CM-medium using one standard concentration of stressing agent. H2O2 represents oxidative stress, CR and SDS represent cell wall stress, and NaCl, KCl, and Sorbitol represent osmotic stress. [file 44154_2026_310_MOESM4_ESM.tif]

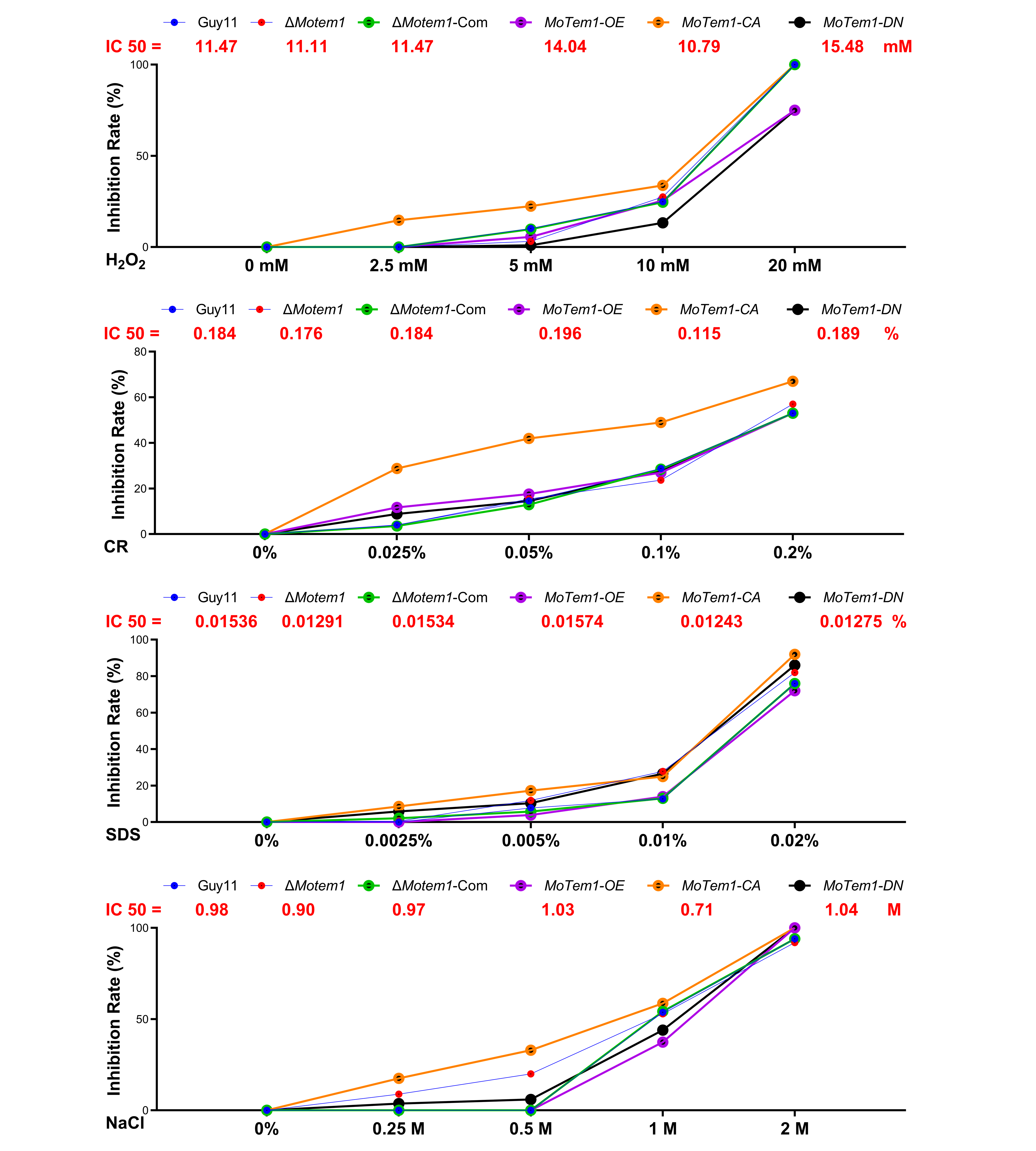

Supplement: Supplementary file 5 — Supplementary Material 5: Fig. S5 Inhibition rates and IC50 values for a range of different concentrations of stressing agents. The half-maximal inhibitory concentration (IC50) was determined to quantify strain sensitivity. Strains were exposed to gradients of H2O2, Congo Red (CR), SDS, and NaCl. After incubation, inhibition rates were calculated. IC50 values were derived by nonlinear regression (GraphPad Prism 10, log[inhibitor] vs. normalized response-Variable slope model). [file 44154_2026_310_MOESM5_ESM.tif]

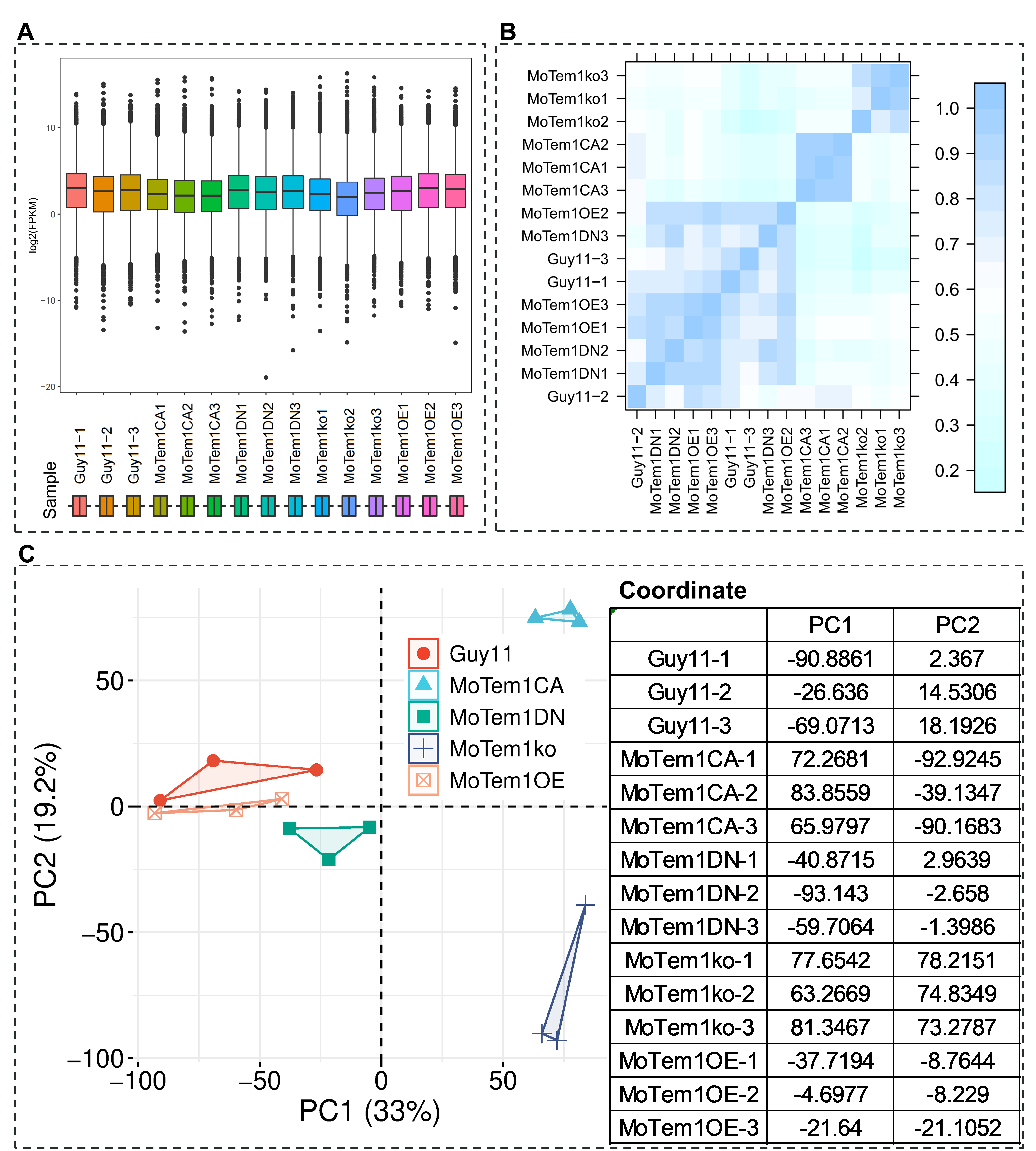

Supplement: Supplementary file 6 — Supplementary Material 6: Fig. S6. Transcriptomic analysis overview. (A) Presents a box plot of transcriptomic data, demonstrating highly consistent median expression levels and distribution ranges across groups, indicating no significant batch-related bias. (B) Shows a correlation heatmap of transcriptomic data, with correlation analysis indicating strong biological reproducibility. (C) A PCA plot, revealing that samples cluster primarily by experimental group, where the OE group and the GUY11 control group exhibit high transcriptional similarity, consistent with phenotypic observations. [file 44154_2026_310_MOESM6_ESM.tif]

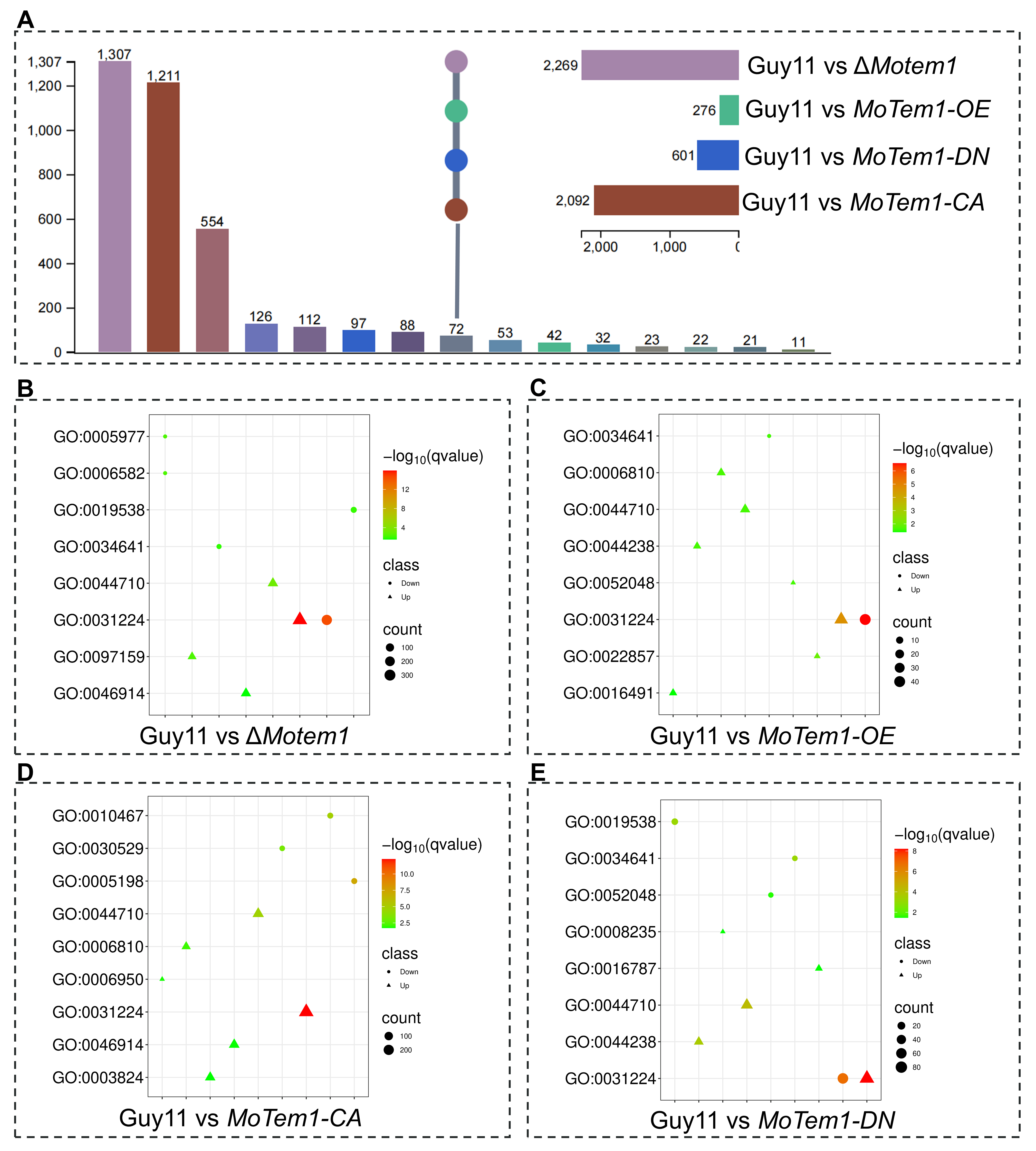

Supplement: Supplementary file 7 — Supplementary Material 7: Fig. S7 Analysis of differentially expressed genes. (A) Shows a differential gene count chart with a similar function as a Venn diagram, showing 72 shared differentially expressed genes (DEGs) compared to Guy 11 (WT). (B-E) Displays GO classification analysis for the DEGs of the four strains (⊿ Motem1, MoTem1-OE, MoTem1-CA, and MoTem1-DN). [file 44154_2026_310_MOESM7_ESM.tif]

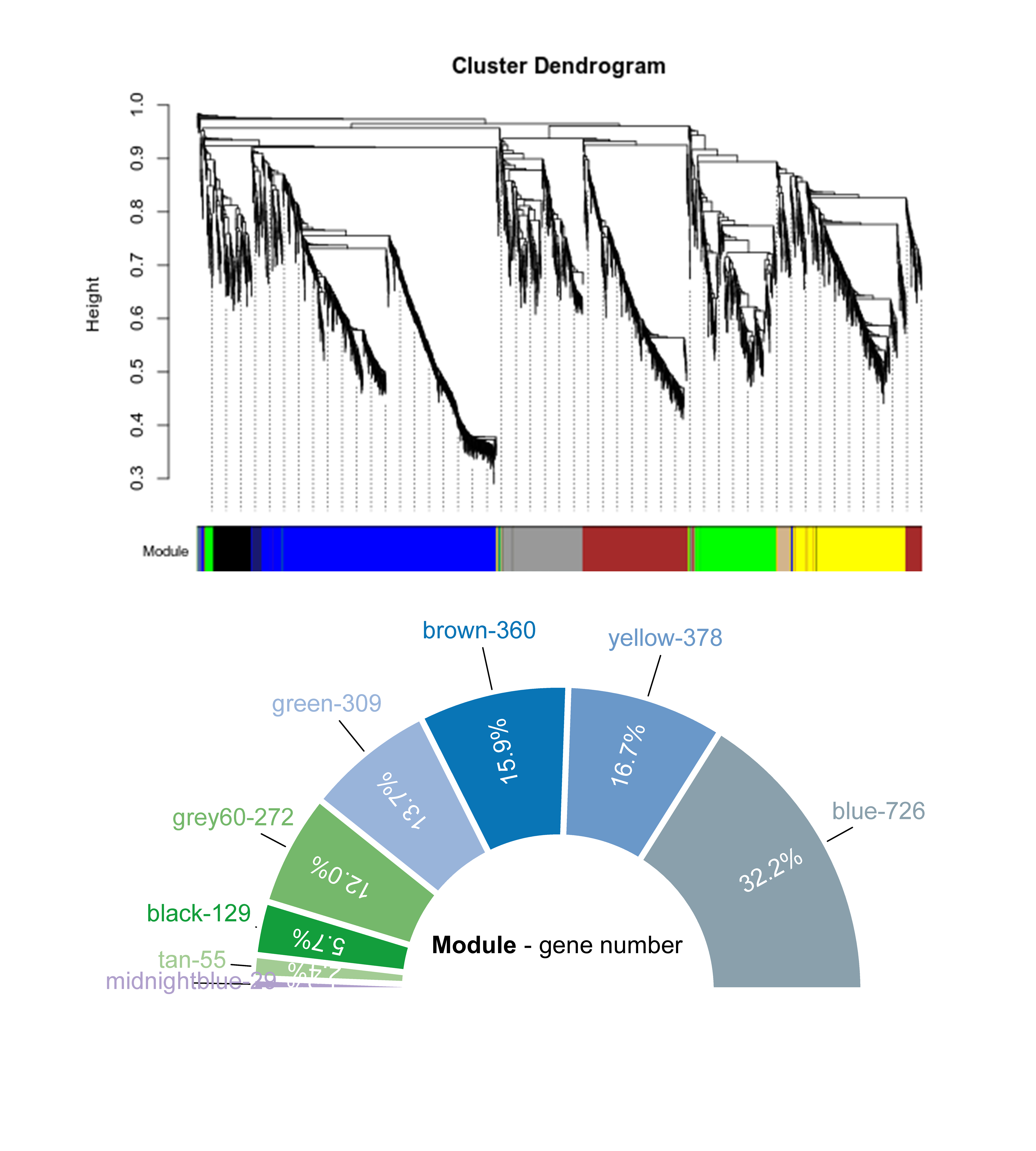

Supplement: Supplementary file 8 — Supplementary Material 8: Fig. S8 Weighted gene co-expression network analysis (WGCNA). Eight distinct modules, named by different colors, were identified. [file 44154_2026_310_MOESM8_ESM.tif]

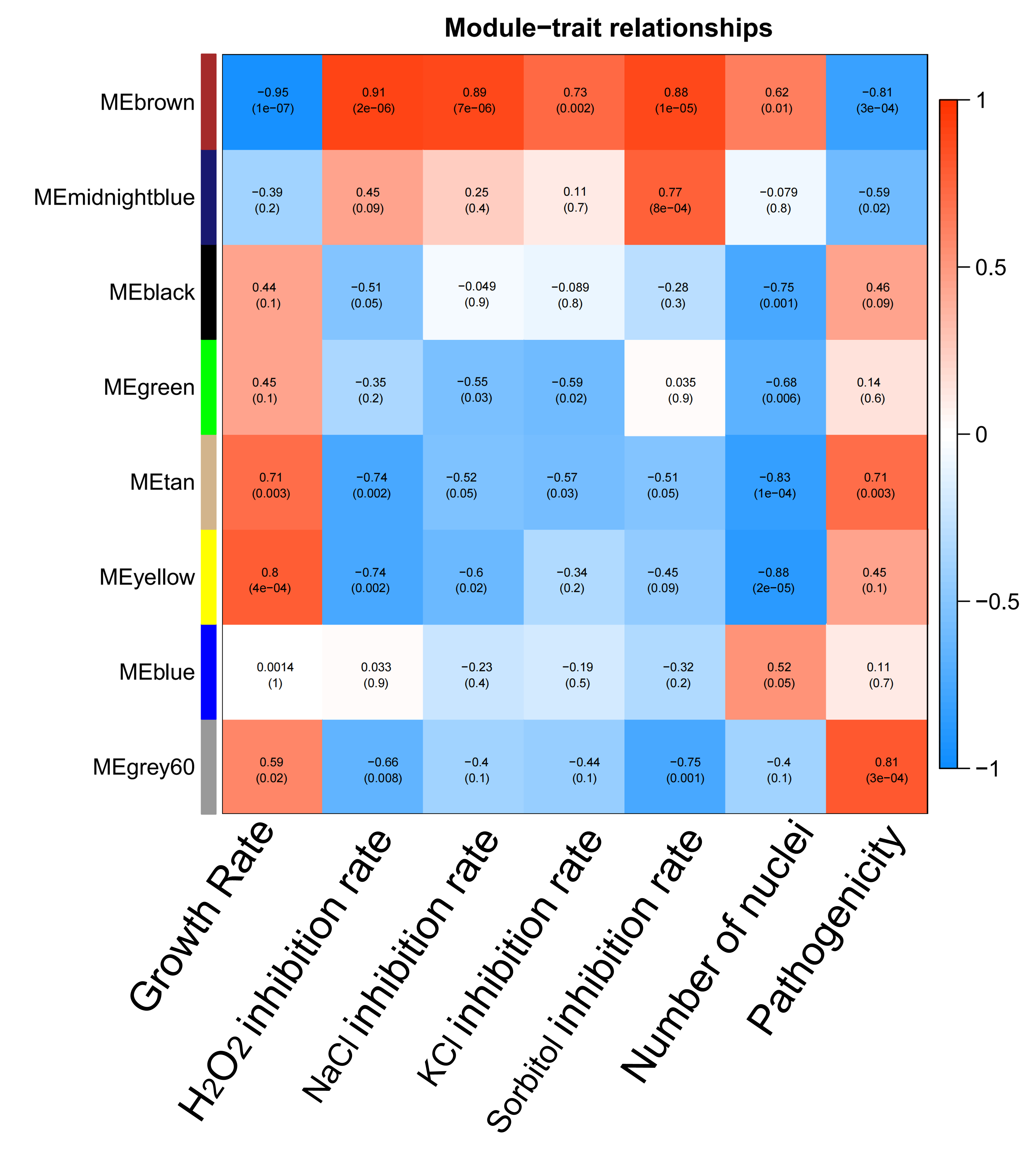

Supplement: Supplementary file 9 — Supplementary Material 9: Fig. S9 Module-trait relationships found by the weighted gene co-expression network analysis (WGCNA). Note especially that the Metan module is strongly correlated with growth rate and pathogenicity. [file 44154_2026_310_MOESM9_ESM.tif]

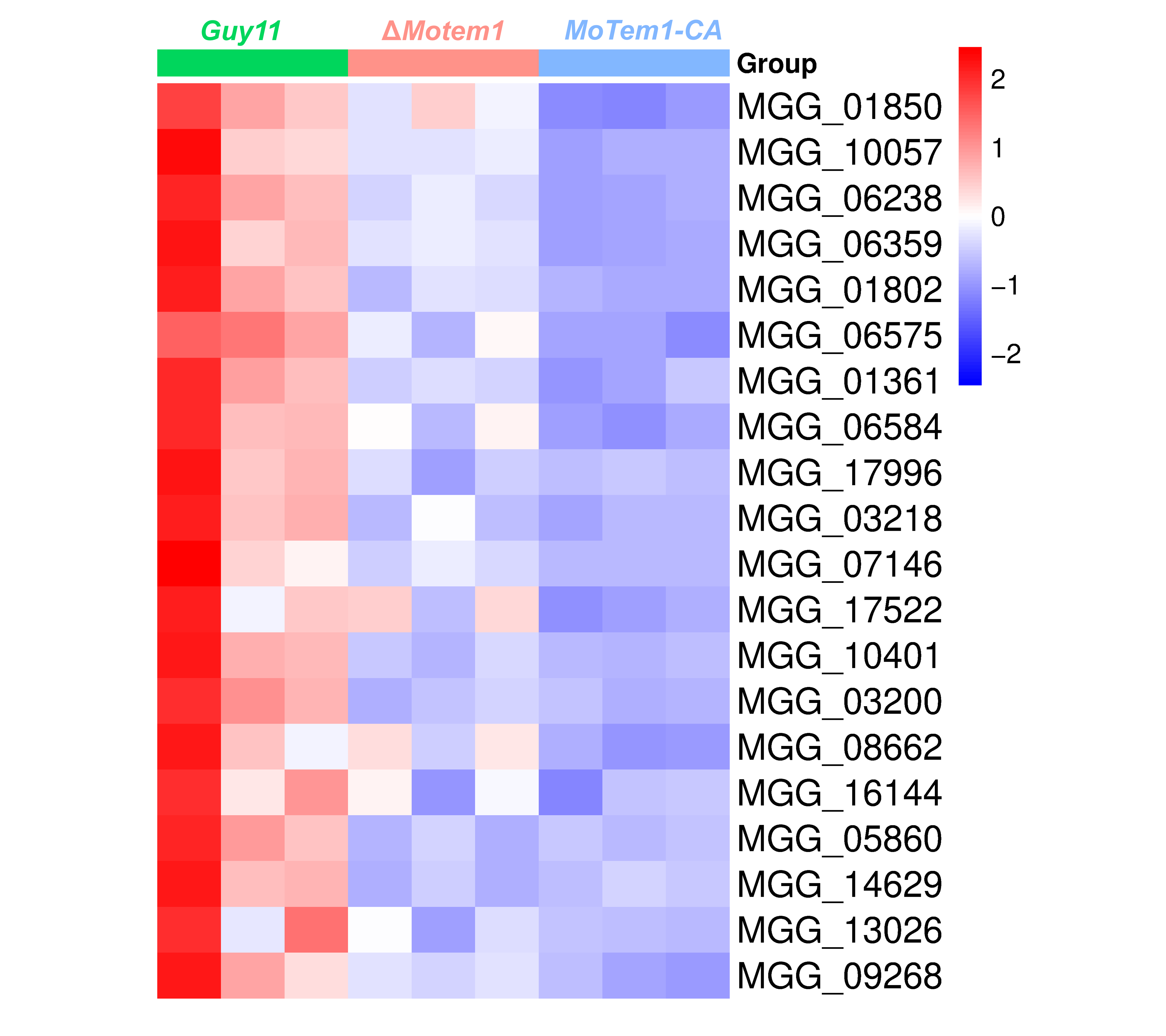

Supplement: Supplementary file 10 — Supplementary Material 10: Fig S10 the FPKM values of the top 20 genes in MeTan for KO and MoTem1-CA strains. Note especially that the chitin synthase MoCHS1 (MGG_01802) is among the genes characterizing the difference between Guy11, △ Motem1, and MoTem1-CA, contributing to the WGCNA identification of the MeTan module strongly correlated with growth rate and pathogenicity. [file 44154_2026_310_MOESM10_ESM.tif]

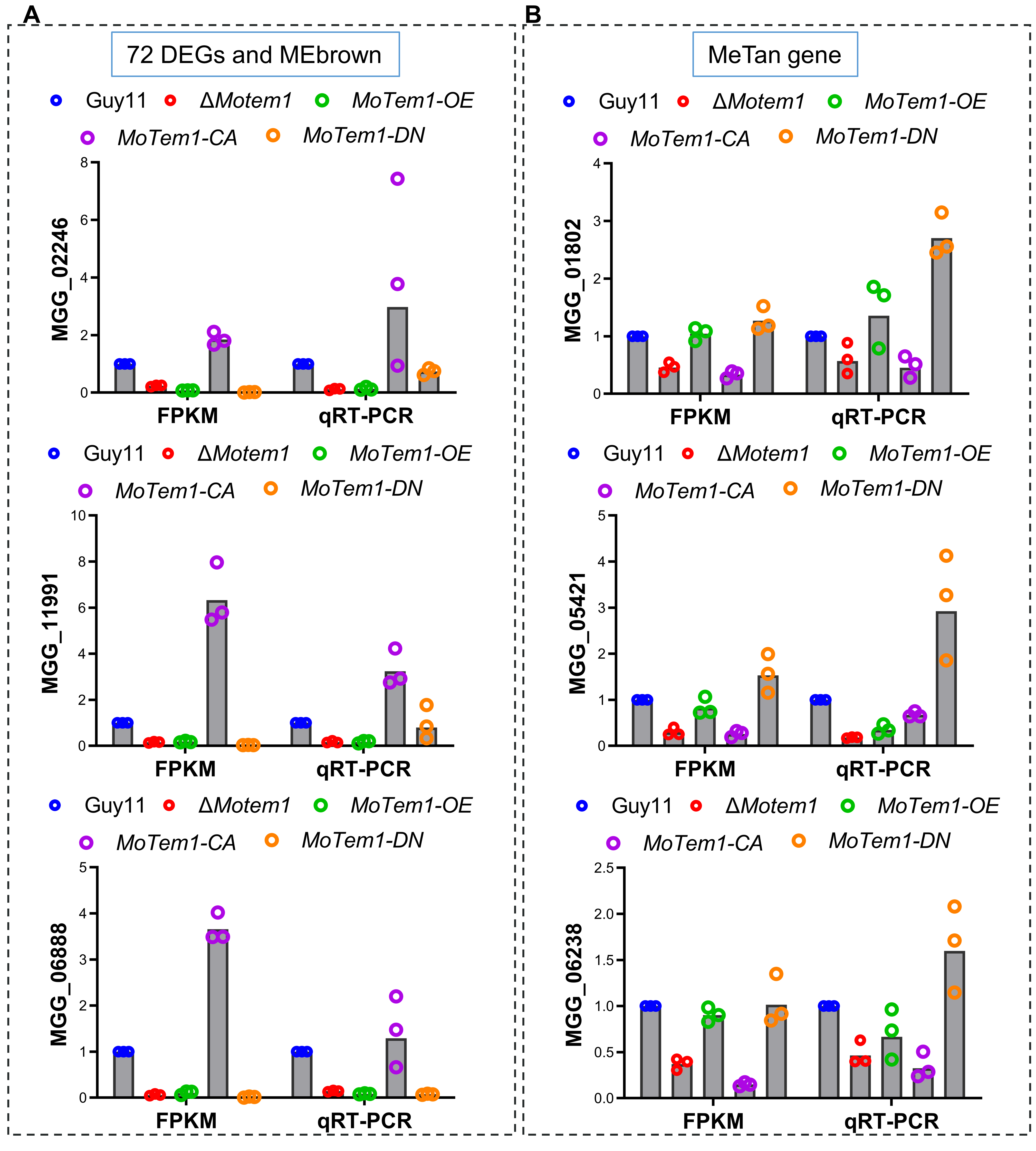

Supplement: Supplementary file 11 — Supplementary Material 11: Fig S11 RT-qPCR validation of RNAsec values for some of the 72 DEGs common to all strains. (A) Three selected genes for MeBrown correlate well. (B) Three selected genes for MeTan correlate well. The MoChs1 (MGG_01802) is one of the genes. [file 44154_2026_310_MOESM11_ESM.tif]

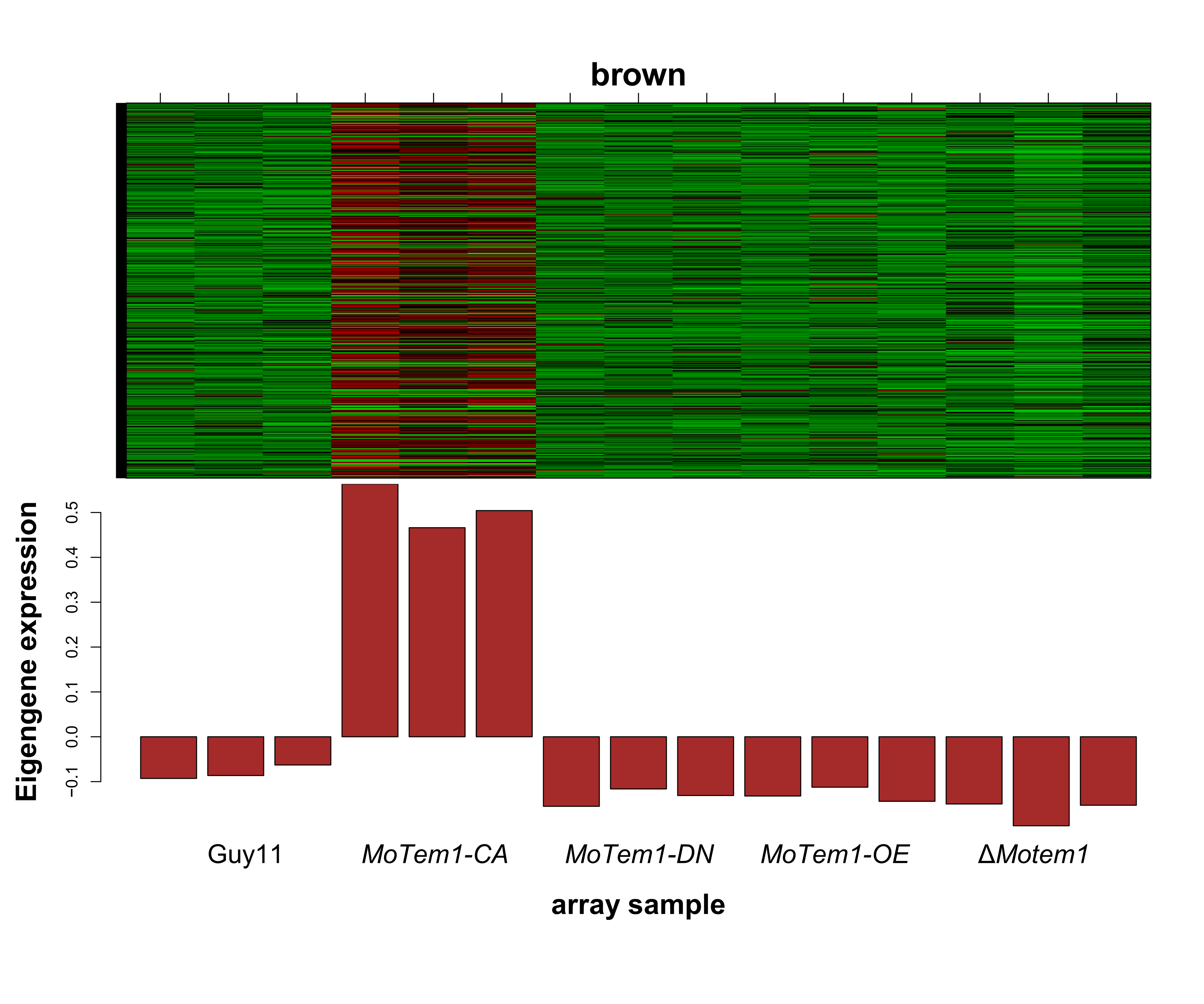

Supplement: Supplementary file 12 — Supplementary Material 12: Fig. S12. Association between the MeBrown module and growth rate. The heatmap shows gene expression in different strains, where green indicates low expression and red indicates high expression, illustrating gene expression profile differences among the samples. [file 44154_2026_310_MOESM12_ESM.tif]

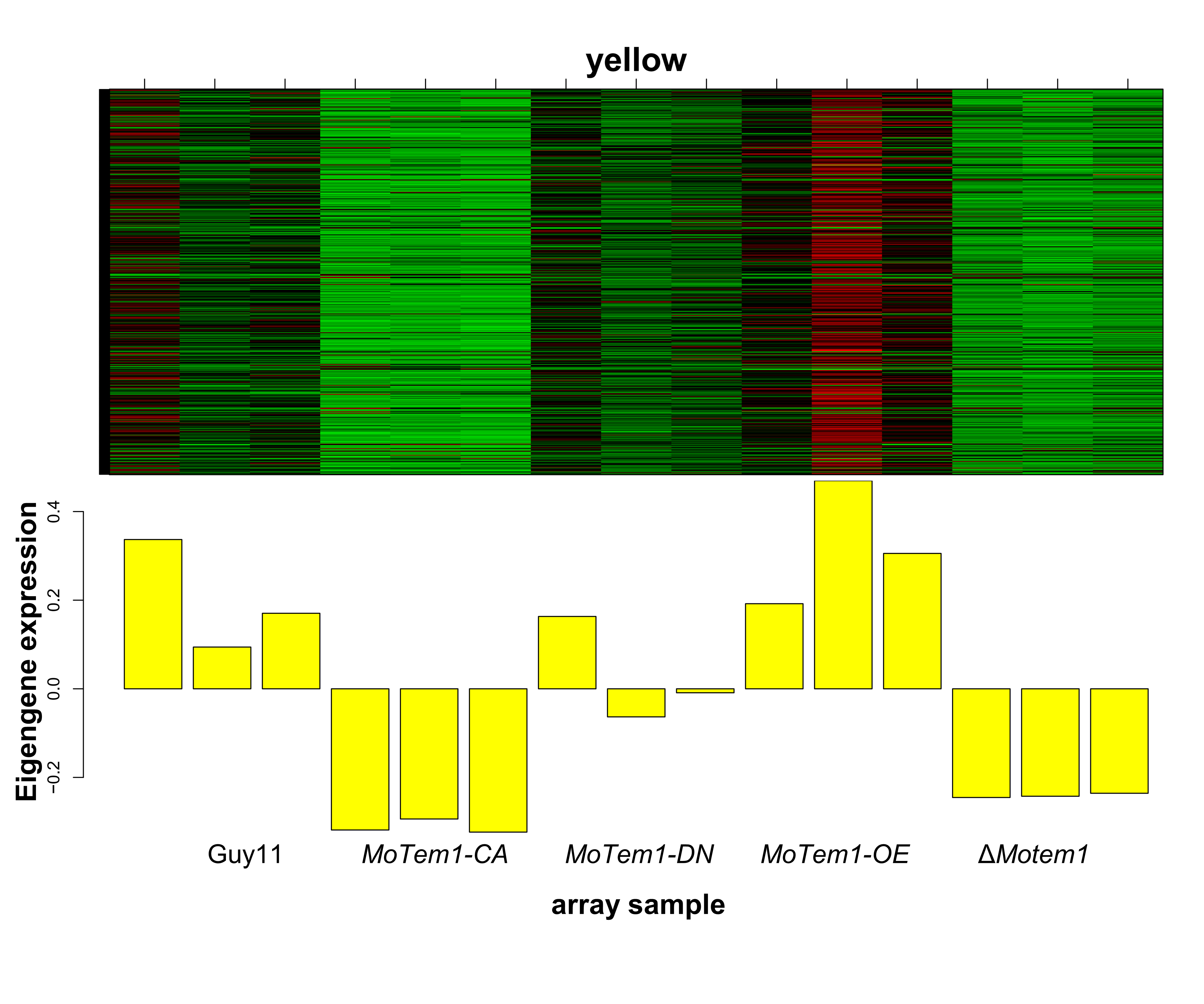

Supplement: Supplementary file 13 — Supplementary Material 13: Fig. S13. Association between the MeYellow module and growth rate. The heatmap shows gene expression in different strains, where green indicates low expression and red indicates high expression, illustrating gene expression profile differences among the samples. [file 44154_2026_310_MOESM13_ESM.tif]

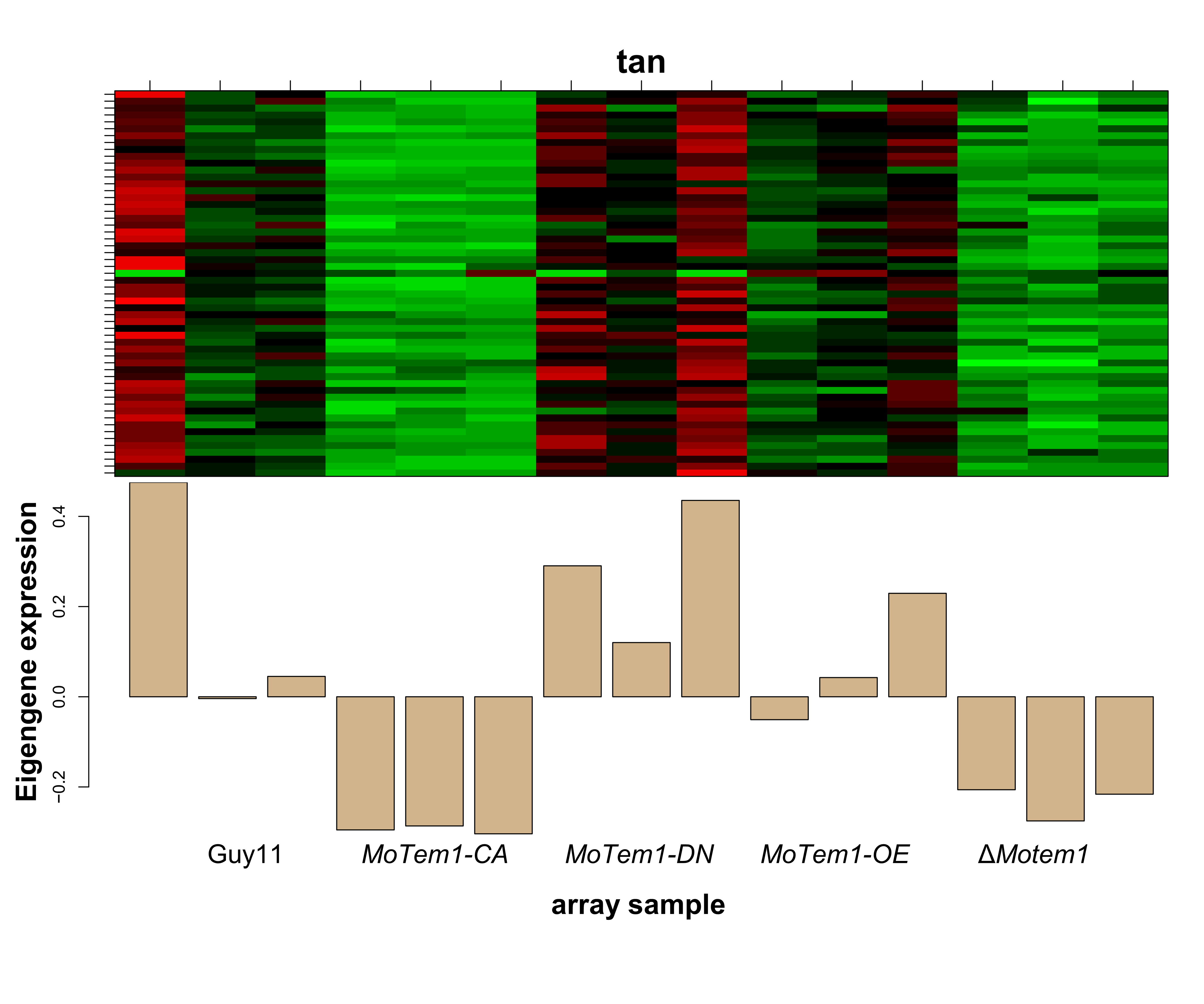

Supplement: Supplementary file 14 — Supplementary Material 14: Fig. S14 Association between the MeTan module and pathogenicity. The heatmap shows gene expression in different strains, where green indicates low expression and red indicates high expression, illustrating gene expression profile differences among the samples. [file 44154_2026_310_MOESM14_ESM.tif]

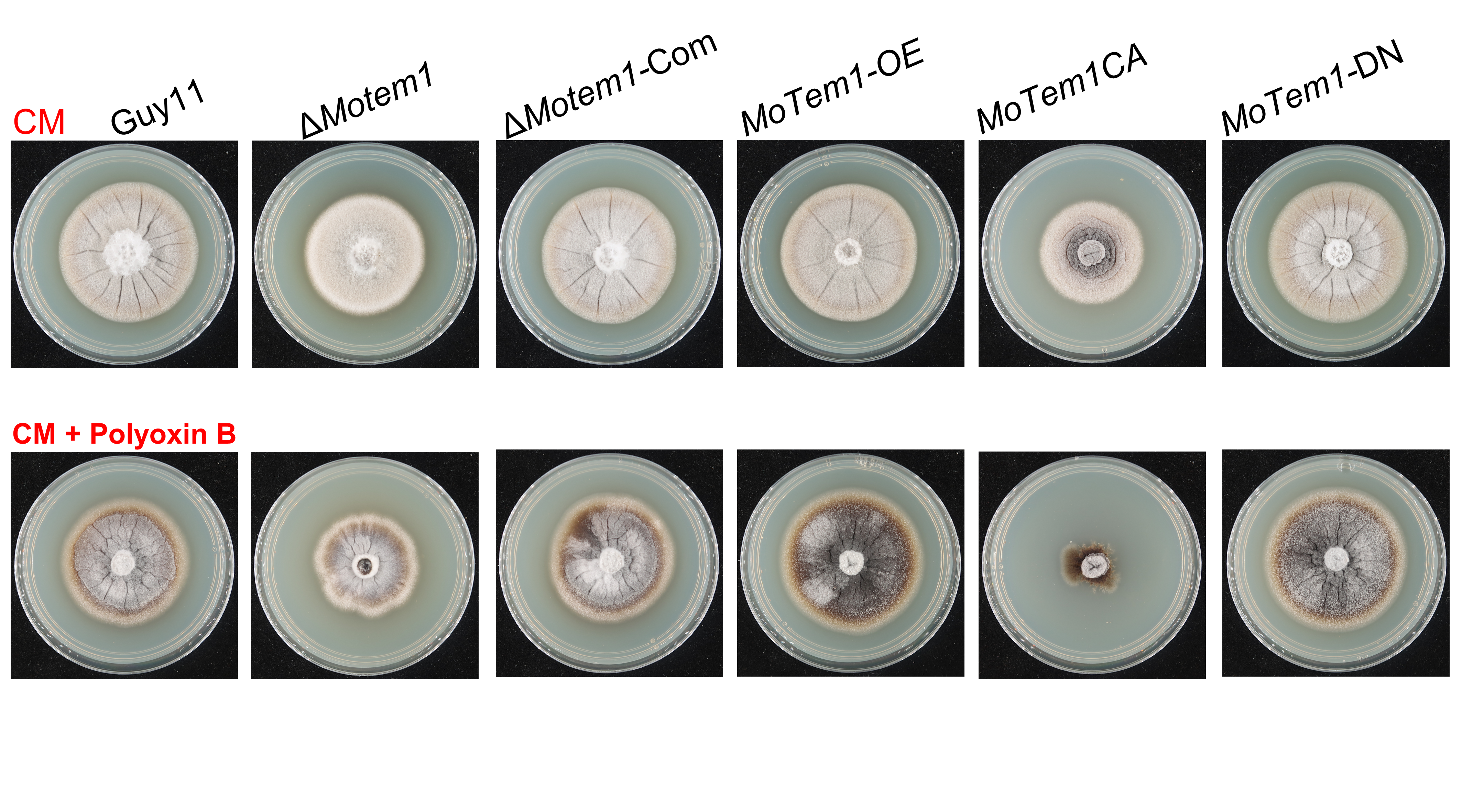

Supplement: Supplementary file 15 — Supplementary Material 15: Fig S15 Colony phenotypes on CM and on CM + polyoxin B. Polyoxin B is a chitin synthase inhibitor, with a working concentration of 100 μg/mL. [file 44154_2026_310_MOESM15_ESM.tif]
